# Electrical Conduction System Remodeling in Streptozotocin-Induced Diabetes Mellitus Rat Heart

**DOI:** 10.3389/fphys.2019.00826

**Published:** 2019-07-08

**Authors:** Yu Zhang, Yanwen Wang, Joseph Yanni, Mohammed Anwar Qureshi, Sunil Jit R. J. Logantha, Sarah Kassab, Mark R. Boyett, Natalie J. Gardiner, Hong Sun, Frank Christopher Howarth, Halina Dobrzynski

**Affiliations:** ^1^Division of Cardiovascular Sciences, School of Medical Sciences, University of Manchester, Manchester, United Kingdom; ^2^Department of Physiology, Xuzhou Medical University, Xuzhou, China; ^3^Department of Physiology, College of Medicine and Health Sciences, United Arab Emirates University, Al Ain, United Arab Emirates

**Keywords:** type I diabetes, arrhythmias, cardiac conduction system, ion channels, calcium handling protein, gap junction channels, remodeling

## Abstract

Cardiovascular complications are common in type 1 diabetes mellitus (TIDM) and there is an increased risk of arrhythmias as a result of dysfunction of the cardiac conduction system (CCS). We have previously shown that, *in vivo*, there is a decrease in the heart rate and prolongation of the QRS complex in streptozotocin-induced type 1 diabetic rats indicating dysfunction of the CCS. The aim of this study was to investigate the function of the *ex vivo* CCS and key proteins that are involved in pacemaker mechanisms in TIDM. RR interval, PR interval and QRS complex duration were significantly increased in diabetic rats. The beating rate of the isolated sinoatrial node (SAN) preparation was significantly decreased in diabetic rats. The funny current density and cell capacitance were significantly decreased in diabetic nodal cells. Western blot showed that proteins involved in the function of the CCS were significantly decreased in diabetic rats, namely: HCN4, Ca_v_1.3, Ca_v_3.1, Cx45, and NCX1 in the SAN; RyR2 and NCX1 in the atrioventricular junction and Cx40, Cx43, Cx45, and RyR2 in the Purkinje network. We conclude that there are complex functional and cellular changes in the CCS in TIDM. The changes in the proteins involved in the function of this electrical system are expected to adversely affect action potential generation and propagation, and these changes are likely to be arrhythmogenic.

## Introduction

The cardiac conduction system (CCS) is a network of specialized myocytes with unique molecular, anatomical, and functional properties that enable them to function as the electrical system of the heart. The CCS includes the sinoatrial node (SAN), atrioventricular junction (AVJ), bundle branches (BB), and Purkinje fibers (PF) (Stephenson et al., 2017). The cells within the CCS do not rely on a single pacemaker mechanism. Instead, there seem to be two separate but closely communicating mechanisms (also termed as “clocks”): a “membrane clock” which consists of ion channels (hyperpolarization-activated cyclic nucleotide-gated channels, mainly HCN4, L-type Ca^2+^ channels, mainly Ca_v_1.3, and T-type Ca^2+^ channels, mainly Ca_v_3.1 and a “calcium clock” which consists of Ca^2+^ – handling proteins (ryanodine receptor, RyR2, sarcoplasmic reticulum-ATPase, SERCA2a, and Na^+^/Ca^2+^ exchanger, and NCX1). The cells of the CCS communicate via gap junctional channels made of connexins (Cx40, Cx43, and Cx45) (Monfredi et al., 2013).

Cardiovascular complications are the major cause of morbidity and mortality in diabetic patients. Type I diabetes mellitus (TIDM) is associated with cardiovascular complications, including cardiac arrhythmias, QT interval prolongation and sudden cardiac death (Nobe et al., 1990). Notably, research has demonstrated that AV node block (Rubler et al., 1975) and bradyarrhythmias (Grimm et al., 1990) are significantly high in the diabetes mellitus (DM) population.

We have previously shown that there are reductions in *in vivo* heart rate and QRS complex prolongation in the streptozotocin (STZ) rat model of TIDM (Howarth et al., 2005). Cardiac remodeling and altered expression of ion channels may be involved in setting the cardiac rate and rhythm (Movahed, 2007). We have previously shown that TIDM affects a range of mRNA expression in the diabetic SAN (NCX1, Cx45, Ca_v_3.1, and HCN4) (Ferdous et al., 2016; Huang et al., 2017).

The aim of this study was to investigate the intrinsic properties of the *ex vivo* CCS and the key proteins that are involved in the CCS pacemaker mechanisms using our well-established STZ model of TIDM. Our aim was also to determine if there is any evidence of fibrosis and apoptosis in the CCS in this model of TIDM.

## Materials and Methods

Procedures were carried out in accordance with the regulations of the United Kingdom Animals (Scientific Procedures) Act 1986 and/or United Arab Emirates. 43 Wistar male rats aged 16 weeks were used. 21 rats between 6 and 8 weeks were injected intraperitoneally with STZ (S0130, Sigma, 60 mg/kg) to induce diabetes as described previously (e.g., Howarth et al., 2008; Newton et al., 2017). 22 age-matched rats were used as controls. Electrocardiogram (ECG) recordings to investigate the intrinsic function of the CCS were performed in Langendorff perfused hearts. Extracellular potential recordings to investigate the beating rate were performed in SAN preparations. Patch clamp recordings were performed on enzymatically isolated SAN cells to study funny current (*I*_f_). Immunohistochemistry and/or Western Blotting were performed to measure protein expression. Data represent mean ± SEM. Statistical analyses were performed using GraphPad Prism7 software. The *t*-test and one-way ANOVA were used to compare the mean values and calculate *P* values; *P* < 0.05 was considered statistically significant.

### *Ex vivo* Electrocardiogram Recording

*Ex vivo* experiments were conducted in isolated Langendorff-perfused hearts from 4 STZ and 4 control animals as previously described (D’Souza et al., 2017). The STZ and control rats were weighed and then euthanized by cervical dislocation following which hearts were dissected in Tyrode solution containing: 100 mM NaCl, 4 mM KCl, 1.2 mM MgSO_4_, 1.2 mM KH_2_PO_4_, 1.8 mM CaCl_2_, 25 mM NaHCO_3_ and 10 mM glucose, pH 7.4. The solution was bubbled with 95% O_2_ and 5% CO_2_. Hearts were cannulated and perfused with Tyrode solution. The recording electrodes interfaced with a Neurolog system (Digitimer). Low-pass, and high-pass filters were adjusted to optimize the signal-to-noise ratio. The *ex vivo* ECG was continuously recorded on a PC with a PowerLab and LabChart V7 software (ADInstruments). The ECGs were recorded using electrodes positioned at the right atrium and ventricular apex. The SAN function was assessed by the measurement of the RR interval (from which the intrinsic heart rate was calculated). The function of the AVJ was assessed by measurement of the PR interval and the Wenckebach cycle length (measured using a suitable pacing protocol). The function of the His-Purkinje system was assessed by the measurement of the QRS duration. The QT duration was measured as an index of ventricular action potential duration.

### Electrophysiology of the Isolated SAN Preparation

The beating rate of the isolated SAN preparation was determined using extracellular potential recording as previously described (Yamamoto et al., 1998). The rats (4 STZ and 4 controls) were weighed and then euthanised by cervical dislocation following which a right atrial preparation encompassing the SAN was rapidly dissected in Tyrode solution (described above). The preparation was superfused with 37°C Tyrode solution at a flow rate of 10 ml/min and extracellular potentials were recorded using bipolar electrodes 100 μm in diameter. The recording electrodes interfaced with a Neurolog system (Digitimer) with low-pass and high-pass filters adjusted to optimize the signal-to-noise ratio. The extracellular potentials were continuously recorded on a PC with a PowerLab and LabChart V7 software (ADInstruments). The effect of CsCl on the beating rate was then studied. The superfusing solution was changed to Tyrode solution containing 2 mM CsCl. After 20 min of treatment, the beating rate was recorded for 5 min. The preparation was then washed of CsCl for 20 min. After 20 min, the superfusing solution was changed to Tyrode solution containing 2 μM ryanodine for 20 min. The preparation was then washed of ryanodine for 20 min. The calculated rate was averaged over 200 beats.

### Isolation of SAN Cells and Patch-Clamp Electrophysiology

Three STZ and 4 control rats were euthanised by cervical dislocation. After quick removal of the heart, the SAN tissue was dissected and strips of nodal tissue were dissociated into single cells by an enzymatic and mechanical procedure as previously described (D’Souza et al., 2017). The enzyme solution contained collagenase IV (224 U ml^–1^, Worthington, OH, United States), elastase (1.42 U ml^–1^, Sigma-Aldrich), and protease (0.45 U ml^–1^ Sigma-Aldrich). *I*_f_ was recorded using a patch electrode in whole-cell mode during superfusion of a Tyrode solution containing (in mM): 140 NaCl, 5.4 KCl, 1.8 CaCl_2_, 1 MgCl_2_, 5 HEPES-NaOH, 10 D-glucose, pH 7.4. BaCl_2_ (1 mM), and MnCl_2_ (2 mM) were added to avoid contamination from other ionic currents. The bath temperature was maintained at 35 ± 0.5°C. The pipette solution contained (in mM): 130 K-aspartate, 10 NaCl, 2 CaCl_2_ (pCa = 7), 2 MgCl_2_, 10 HEPES, 5 EGTA, 2 ATP(Na_2_), 0.1 GTP, 5 creatine phosphate, pH 7.2. To obtain current densities, currents were measured during steps to the range -35 to -125 mV from a holding potential of -35 mV and normalized to cell capacitance. Data were acquired at 1 kHz using an Axopatch 200 amplifier and pClamp 8 (Molecular Devices, Sunnyvale, CA, United States). Data were analyzed off-line using Clampfit 10 (Molecular Devices), Origin 8 (Origin Lab Corp., Northampton, MA, United States) and GraphPad Prism version 6 (GraphPad Software, Inc.). 33 nodal cells from STZ rats and 29 nodal cells from control rats were analyzed.

### Western Blot

Different proteins (see Supplementary Table S1) were investigated in different regions of the heart by Western blot using previously described methods (D’Souza et al., 2017). 5 STZ and 5 control rats were used for these experiments. SANs (center and periphery) were dissected from the junction of the superior vena cava and right atrium, which also contain the nodal artery and a small amount of the right atrial myocardium. AVJs were dissected from the junction between ventricular and atrial septum, which mainly contained HIS and penetrating bundle and compact node and a small amount of the surrounding working myocardium. Left Purkinje fibers (LPFs) (free running Purkinje network) were dissected from the surface in the left ventricle. Samples of left atria (LA) and right ventricle (RV) were also dissected. Protein lysate was obtained by homogenizing snap frozen tissue samples from different regions of the heart using an MP FastPrep-24 5Gand 2 ml tubes containing FastPrep metal bead lysing matrix (1.4 mm) in RIPA buffer (Sigma Aldich). Total protein concentration was estimated using Bradford protein assay against a standard curve of bovine serum albumin (BSA; 0–0.4 mg/ml) following which samples were denatured by adding 25% SDS-sample buffer and heating to 80°C for 5 min. SDS-sample buffer contained: 100 mM Tris–HCl, pH 6.8, 25% (v/v) glycerol, 10% (v/v) SDS, 10% (v/v) β-mercaptoethanol, and 0.1% (w/v) bromophenol blue. Samples were loaded onto stain-free SDS-polyacrylamide gels (Bio-Rad) with PreSciccion Plus (Bio-Rad) protein standards and run at 60 mV for ∼45 min in SDS running buffer (25 mM Tris, 192 mM glycine, 0.1% SDS). Stain-free gels were imaged using ChemiDoc MP and then transferred to PVDF (polyvinyl difluoride) membranes using a Trans-Blot Turbo transfer system (Bio-Rad) at 15 V/0.3 mA for 15 min. PVDF membranes (activated by 100% ethanol) and thick filter paper were pre-wet in transfer buffer: 5× Trans-Blot Turbo transfer buffer (Bio-Rad), 20% (v/v) ethanol, and 60% (v/v) ethanol. The traditional wet transfer was used for the target proteins with high molecular weight (≥150 kDa). Stain-free gels were transferred to PVDF membranes using a wet transfer system (Bio-Rad) at 100 mA for 45 min. PVDF membranes (activated by 100% ethanol) and thick filter paper were pre-wet in transfer buffer: 20% (w/v) methanol; 25 mM Tris; 192 mM glycine; pH 8.3. Successfully transferred PVDF membranes were confirmed by using the ChemiDoc MP. PVDF membranes were washed in TBS for 10 min and then blocked in milk-TBS-Tween (5% w/v non-fat dried Marvel milk and 1% w/v BSA, 0.1% v/v TBS and Tween 20) for 60 min at room temperature with gentle rocking. The membranes were then incubated with primary antibodies (Supplementary Table S1) for 60 min at room temperature with gentle rocking. Following three 10 min washes in TBS-Tween 20, membranes were then probed with horseradish peroxidase (HRP)-linked secondary antibody (Supplementary Table S2) for a further 60 min at room temperature with gentle rocking. Membranes were then washed three times for 10 min in TBS-Tween to remove unbound secondary antibody. Chemiluminescence was achieved by the addition of Clarity Western ECL substrate (Bio-Rad) in a 1:1 ratio for 10 min in the dark. Membranes were then imaged with the ChemiDoc MP. STZ and control samples were run on the same gel to ensure identical exposure conditions. The chemiluminescent signal intensity was normalized to the relative quantification of the corresponding intensity of β-actin. The membrane images are shown in Supplementary Figures S7–S21.

### Immunohistochemistry and TUNEL Staining

Five hearts from STZ and 5 hearts from control rats were removed and flash frozen with liquid N_2_ and stored at −80°C until the tissue was processed. The frozen hearts were serially cryosectioned at 18 μm from the posterior to anterior sides in the coronal plane to obtain the four-chamber view. Immunohistochemistry was used to label different regions of the heart using primary antibodies (Supplementary Table S1) as previously described (Yanni et al., 2010). Frozen sections were fixed in 10% neutral buffered formalin, washed in PBS (NaCl, 8 g; KCl, 0.2 g; NaHPO_4_, 1.44 g; KHPO_4_, 0.24 g; deionised water, 1 l), permeabilized in 0.1% Triton X-100 in PBS, washed in PBS, blocked with 3% BSA in PBS and incubated in primary antibodies at 4°C overnight. The following day, after PBS wash, secondary antibodies and TUNEL assay (to some sections) were applied (Supplementary Table S3) for 120 min and washed in PBS. Tissue sections were then mounted with an anti-fade medium for fluorescence (Vectashied; Vector Lab, Peterborough, United Kingdom), covered with coverslips and sealed with nail varnish. Confocal images were acquired using a laser-scanning microscope (Zeiss LSM 5 PASCAL). Images were acquired using the following conditions: 488 nm excitation and 505–530 nm emission for FITC and TUNEL, and 543 nm excitation and >560 nm emission for Cy3.

### Statistical Analysis

Statistical analysis was carried out using GraphPad Prism version 6 or 7 (GraphPad Software). One-way ANOVA (followed by *post hoc* test) was used for data normally distributed and a non-parametric test (Mann-Whitney test) was used for the data not normally distributed. Two-way ANOVA (followed by Bonferroni *post hoc* tests) was used for data amongst groups and different data sets. *P* < 0.05 was regarded as statistically significant.

## Results

### Changes in Heart Rhythm in STZ Rat

**FIGURE 1:**
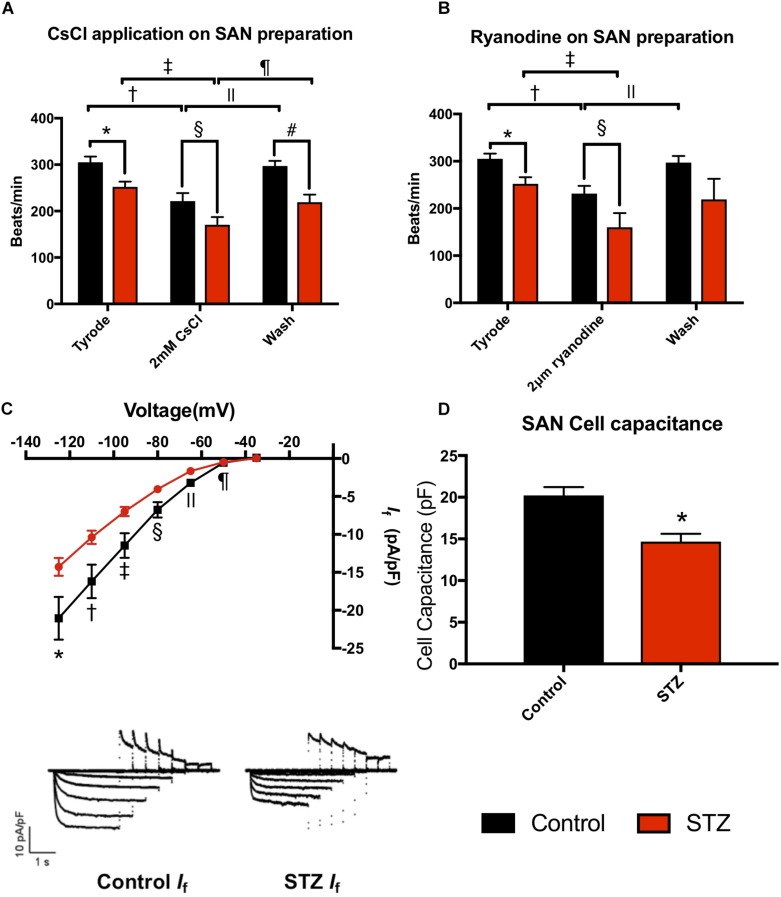
Effect of STZ-induced type I diabetes mellitus on heart rhythm. **(A)** Mean heart rate recorded from the isolated SAN in normal Tyrode’s solution, on perfusing with 2 mM CsCl and on wash off of CsCl. Data from control (black bars; *n* = 4) and TIDM (red bars; *n* = 4) rats shown. **(B)** Mean heart rate recorded from the isolated SAN in normal Tyrode’s solution, on perfusing with 2 μM and on wash off of ryanodine. Data from control (black bars; *n* = 4) and TIDM (red bars; *n* = 4) rats shown. In **(A,B)**, symbols shown significantly different. **(C)** Mean current-voltage relationship for *I*_f_ recorded from isolated SAN cells from control (black) and TIDM (red) rats. *I*_f_ density is plotted. Control: *n* = 4 rats, *n* = 29 cells. TIDM: *n* = 3 rats, *n* = 33 cells. Symbols show significantly different from corresponding control data. Representative traces of *I*_f_ from control and TIDM rats shown as an inset. **(D)** Mean cell capacitance of SAN cells from control and TIDM rats. Control: *n* = 4 rats, *n* = 29 cells. TIDM: *n* = 3 rats, *n* = 33 cells. ^*^Significantly different from control. Data are means ± SEM; *P* < 0.05.

Experiments were conducted in STZ (*n* = 21) and age matched control rats (*n* = 21) 8 weeks after STZ treatment. The body weight and heart weight of diabetic rats were significantly lower compared to controls (Table 1 and Supplementary Figure S1). The heart weight to body weight ratio and glucose level were significantly increased in diabetic rats (Table 1 and Supplementary Figure S1). The *ex vivo* ECG parameters show significantly prolonged RR interval, PR interval, QT interval and QRS complex (Table 1). The beating rate of the STZ rat SAN was slower compared to controls (Figure 1). Applying CsCl to the isolated SAN to block the HCN channels and *I*_f_ of the membrane clock or ryanodine to incapacitate the Ca^2+^ clock slowed pacemaking in STZ and control rats, demonstrating that both pacemaker mechanisms are operative (Figures 1A,B). The CsCl induced decrease in heart rate, which is smaller in the STZ rat hearts and this is suggestive of a decrease of *I*_f_ in TIDM. Whole cell patch-clamp recordings from isolated SAN cells showed that the cell capacitance and density of *I*_f_ were reduced in STZ rat nodal cells (Figures 1C,D and Supplementary Table S4). Arrhythmias were observed in the STZ rat SAN on application of ryanodine, Supplementary Figure S2.

**TABLE 1:** Effect of STZ-induced type I diabetes mellitus on heart rhythm.

	**Control**	**STZ**
**Characteristic of rat model**		
Body weight (g)	322.50±22.4	191.00±37.4*
Heart weight (g)	1.18±0.12	0.85±0.14^†^
Heart/body weight ratio	3.59±0.32	4.19±0.35^‡^
Blood glucose (mg/dl)	101.91±10.5	533.33±45.1^§^
***Ex vivo* ECG parameter**		
RR interval (s)	0.196±0.009	0.238±0.022^||^
PR interval (s)	0.039±0.011	0.047±0.013^¶^
QT interval (s)	0.052±0.009	0.068±0.005^#^
QRS complex (s)	0.0124±0.0003	0.0154±0.0002^**^

(A) General characteristics of STZ-induced diabetic rats (*n* = 21) vs. age-matched controls (*n* = 22). (B) *Ex vivo* ECG parameter of STZ-induced type I diabetic rats (*n* = 4) compared to controls (*n* = 4). ^*^Significantly different in body weight, ^†^Significantly different in heart weight. ^‡^Significantly different in heart/body weight ratio. ^§^Significantly different in blood glucose. ^||^Significantly different in RR interval. ^¶^Significantly different in PR interval. ^#^Significantly different in QT interval. ^∗∗^Significant differently in QRS complex. Data are means ± SEM; *P* < 0.05.

### Identification of Different Components of the Cardiac Conduction System in the Rat Heart

**FIGURE 2:**
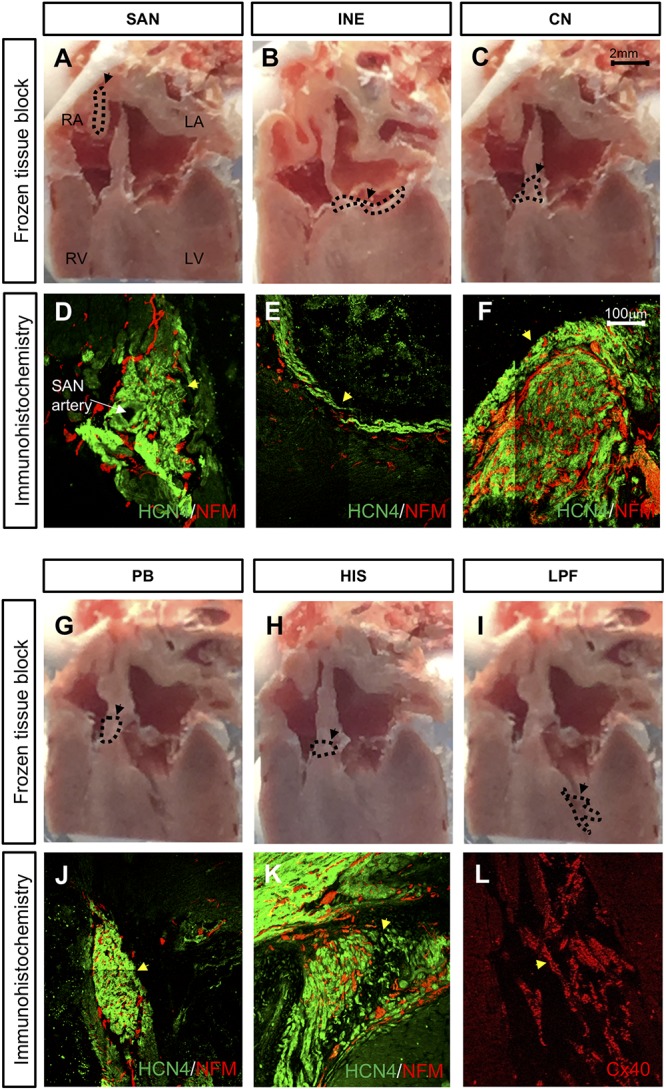
Location of cardiac conduction system (CCS) in control rat hearts (*n* = 4). **(A**,**B**,**C**,**G**,**H**,**I)** Frozen tissue block at the different regions of the CCS. Dashed line highlights the location different regions of the CCS in these images. Bar = 2 mm. **(D**,**E**,**F**,**J**,**K**,**L)** High-magnification images of HCN4 labeling (green), NF-M labeling (red), and Cx40 labeling (red). Bar = 100 μm. CN, compact node; HIS, bundle of His; INE, inferior nodal extension; LPF, left Purkinje fiber; PB, penetrating bundle; SAN, sinoatrial node. Black circle and arrow in A = SAN, black circle and arrow in B = INE, black circle and arrow in C = CN, black circle and arrow in G = PB, black circle and arrow in H = HIS, and black circle and arrow in I = LPF. Yellow arrow in D = SAN, yellow arrow in E = INE, yellow arrow in F = CN, yellow arrow in J = PB, yellow arrow in K = HIS, and yellow arrow in L = LPF.

The SAN is located at the junction of the superior caval vein and right atrium (Figures 2A,D). The SAN is supplied by the sinus node artery (Figure 2D). The AVJ is located at the base of the atrial septum and includes the inferior nodal extension (INE, Figures 2B,E), compact node (CN, Figures 2C,F), penetrating bundle (PB, Figures 2G,J) and bundle of His (HIS, Figures 2H,K). The INE is the extension of the compact node (Figures 2B,E). The CN is an oval shaped structure (Figures 2C,F). The PB (proximal to the CN) is located between the atrial septum and ventricular septum (Figures 2G,J). HIS (dorsal to the CN) is located at the crest of the ventricular septum (Figures 2H,K). All these cardiac conduction tissues express HCN4 (shown in green and are heavily innervated by the sympathetic nerves, which are positively labeled for NF-M shown in red in Figure 2). The PFs run along and cover the endocardial side of the left and right ventricles, forming a network. Figures 2I,L show the location of a part of the left Purkinje fiber (LPF) network. PFs express Cx40 (Figure 2L).

### Changes in Membrane Clock Proteins in STZ Rat Heart

**FIGURE 3:**
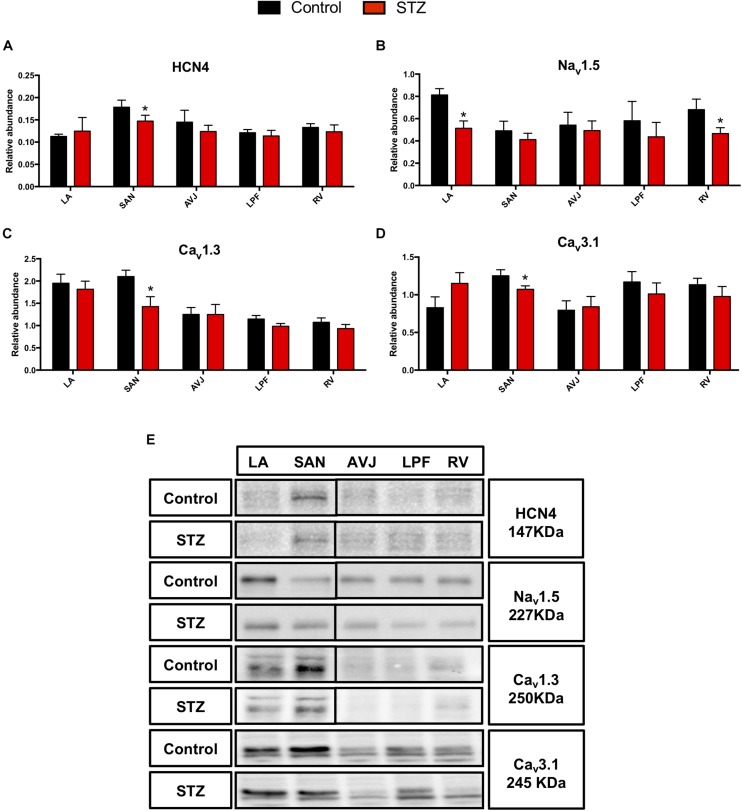
Western blot of HCN4, Na_v_1.5, Ca_v_1.3 and Ca_v_3.1 and in STZ-induced diabetic rats vs. control rats. **(A–D)** Mean relative abundance (normalized to β-actin) of HCN4, Na_v_1.5, Ca_v_1.3, and Ca_v_3.1 in the LA (left atrium), SAN (sinoatrial node), AVJ (atrioventricular junction), LPF (left Purkinje fibers) and RV (right ventricle). **(E)** Western blot of SDS polyacrylamide gel electrophoresis of homogenized tissue samples obtained from STZ-induced type I diabetic hearts and controls labeled for HCN4, Na_v_1.5 Ca_v_1.3, and Ca_v_3.1 proteins. ^*^Significantly different from control. Data are mean ± SEM (*n* = 5 for each group); *P* < 0.05.

The hyperpolarization-activated cyclic nucleotide-gated channel (HCN4), L-type voltage gated Ca^2+^ channel (Ca_v_1.3) and T-type voltage gated Ca^2+^ channel (Ca_v_3.1) are important ion channels involved in the membrane clock. Western blot analysis demonstrates that HCN4 was significantly decreased by 29% in the SAN of diabetic rat heart (Figure 3A). Ca_v_1.3 and Ca_v_3.1 were significantly decreased by 32 and 14% respectively, in the SAN of STZ rat heart (Figures 3C,D).

Na_v_1.5, a voltage-dependent cardiac Na^+^ channel involved in the upstroke of the action potential in atrial and ventricular myocytes, was significantly decreased in the LA by 33% and by 37% in the RV (Figure 3B). Figure 3E shows specific Western blot bands of HCN4, Na_v_1.5, Ca_v_1.3, and Ca_v_3.1 in different regions of the heart.

### Changes in “Ca^2+^ Clock” Proteins in the STZ Rat Heart

**FIGURE 4:**
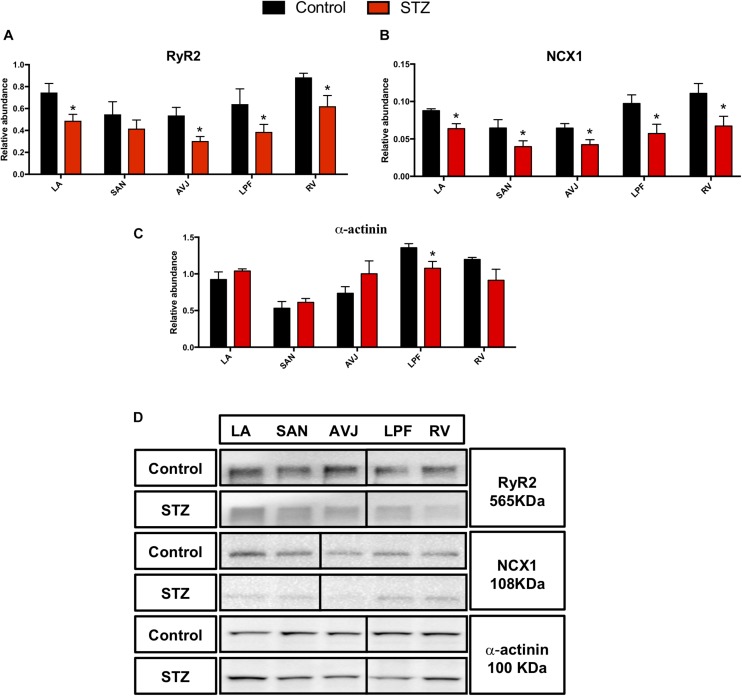
Western blot of RyR2, NCX1, and α-actinin in STZ-induced diabetic rats vs. control rats. **(A–C)** Mean relative abundance (normalized to β-actin) of RyR2, NCX1, and α-actinin in the LA (left atrium), SAN, AVJ, LPF, and RV (right ventricle). **(D)** Western blot of SDS polyacrylamide gel electrophoresis of homogenized tissue samples obtained from STZ-induced type I diabetic rat and controls labeled for RyR2, NCX1, and α-actinin proteins. ^*^Significantly different from control. Data are mean ± SEM (*n* = 5 for each group); *P* < 0.05.

The ryanodine receptor (RyR2) and Na^+^/Ca^2+^ exchanger (NCX1) are important in regulating the Ca^2+^ clock. Western blot analysis demonstrates that RyR2 was significantly decreased in the LA (by 35%), atrioventricular junction (AVJ; by 43%), left Purkinje fibers (LPF; by 40%), and RV (by 30%) in the STZ rat heart compared with controls (Figure 4A). NCX1 was significantly decreased in LA (by 25%), SAN (by 45%), AVJ (by 33%), LPF (by 44%), and RV (by 45%) in the STZ rat heart (Figure 4B).

In addition, we investigated the expression of α-actinin, a cytoskeleton protein, which plays a critical role in maintaining and modulating cell morphology, and elasticity as well as contractility. There was a 21% decrease of α-actinin in the LPF in the STZ rat heart (Figure 4C). Figure 4D shows specific Western blot bands of RyR2, NCX1, and α-actinin in the different regions of the heart.

### Changes in Gap Junction Channel Proteins in the STZ Rat Heart

The gap junction channels provide electrical coupling between cardiac cells (Boyett et al., 2006). The major components of the cardiac gap junctions are connexin40 (Cx40), which is primarily expressed in the atrial muscle and HIS-PF system, connexin43 (Cx43), which is primarily expressed in atrial and ventricular muscle as well as HIS-PF, and connexin45 (Cx45), which is expressed in all the regions of the CCS and ventricular muscle.

**FIGURE 5:**
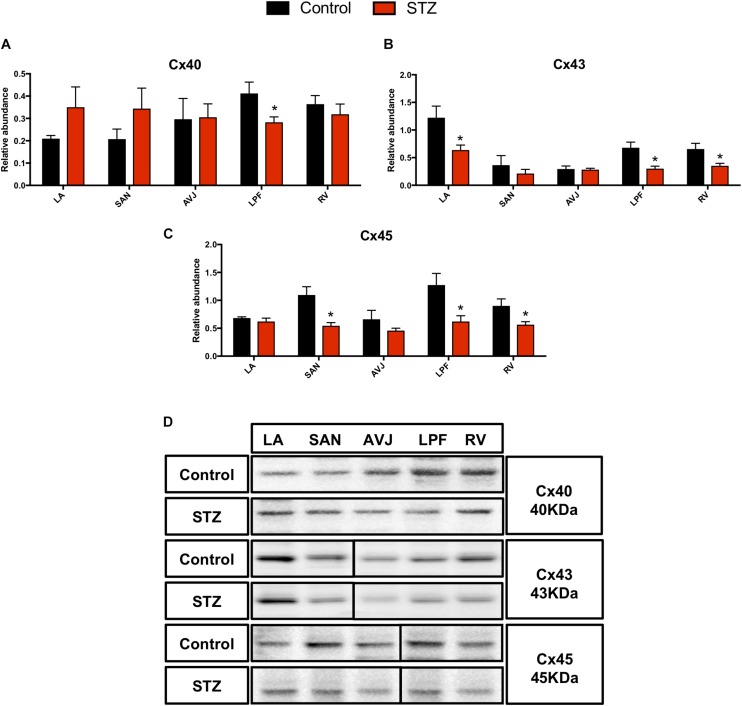
Western blot of Cx40, Cx43, and Cx45 in STZ-induced diabetic rats vs. control rats. **(A–C)** Mean relative abundance (normalized to β-actin) of Cx40, Cx43, and Cx45 in the LA (left atrium), SAN, AVJ, LPF, and RV (right ventricle). **(D)** Western blot of SDS polyacrylamide gel electrophoresis of homogenized tissue samples obtained from STZ-induced type I diabetic rats and controls labeled for Cx40, Cx43, and Cx45 proteins. ^*^Significantly different from control. Data are mean ± SEM (*n* = 5 for each group); *P* < 0.05.

Western blot analysis demonstrated that Cx40 was significantly decreased in the LPF (by 32%) in the STZ rat heart (Figure 5A). Cx43 was significantly decreased in the LA (by 48%), LPF (by 55%), and RV (by 46%) in the STZ rat heart, Figure 5B. Cx45 was significantly decreased in the SAN (by 50%), LPF (by 51%), and RV (by 7%) in the STZ rat heart (Figure 5C). Figure 5D shows specific Western blot bands of Cx40, Cx43, and Cx45 in the different regions of the heart. These proteins were detected at the molecular weights listed on the right of Figure 5D.

Supplementary Figure S4 shows immunolabeling of Cx40, Cx43, and Cx45 in the SAN and LPF in the STZ rat heart and control heart. This figure shows that there was a weaker signal for Cx45 in the SAN from the STZ rat heart (Supplementary Figures S4A,B). There were weaker signals for Cx40 in the LPF (Supplementary Figures S4C–F), Cx43 in the LPF (Supplementary Figures S4G,H), and Cx45 in the LPF in STZ rat heart compared to controls (Supplementary Figures S4I,J).

### Changes in Autonomic Regulation Related Proteins in STZ Rat Heart

**FIGURE 6:**
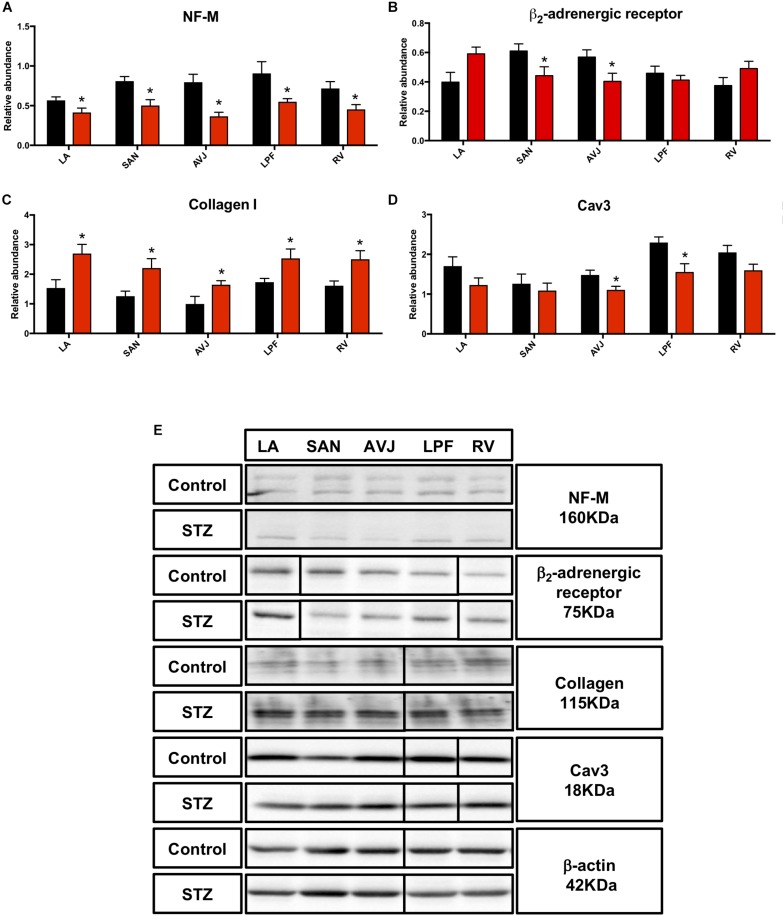
Western blot of NF-M, β_2_-adrenergic receptor, Collagen I, and Cav3 in STZ-induced diabetic rats vs. control rats. **(A)** Mean relative abundance (normalized to β-actin) of NF-M, **(B)** β_2_-adrenergic receptor, **(C)** Collagen I, and **(D)** Cav3 in the LA (left atrium), SAN (sinus node), AVJ, LPF, and RV (right ventricle). **(E)** Western blot of SDS polyacrylamide gel electrophoresis of homogenized tissue samples obtained from STZ-induced type I diabetic rats and control rats labeled for NF-M, β_2_-adrenergic receptor, collagen I, Cav3 proteins, and beta-actin. ^*^Significantly different from control. Data are mean ± SEM (*n* = 5 for each group); *P* < 0.05.

The sympathetic nervous system, as a component of the autonomic nervous system, is responsible for acceleration of cardiac function via the activation of the β-adrenergic receptor and its downstream signaling pathway (Krstacic et al., 2013). A cytoskeletal neurofilament (NF) is one of the major constituents of the axon and its expression level determines axonal function and size, which is thought to indirectly impact the heart rate (Pierson et al., 2003). Western blot analysis demonstrated that the middle NF (NF-M) was significantly decreased in the LA (by 27%), SAN (by 37%), AVJ (by 54%), LPF (by 40%), and RV (by 37%) in the STZ rat heart (Figure 6A). The β_2_-adrenergic receptor was significantly decreased in the SAN (by 28%) and AVJ (by 29%) in the STZ rat heart (Figure 6B). Figure 6E shows specific Western blot bands of NF-M and β_2_-adrenergic receptor in the different regions of the heart.

### Changes in Collagen I, Caveolin3 Expression, and TUNEL Staining in STZ Rat Heart

Collagen I, an extracellular structural protein, is the main component of connective tissues in the heart (Bishop and Laurent, 1995), whereas caveolin3 (Cav3) is a structural protein in heart cells and influences the activity of ion channels (Vassilopoulos et al., 2010). Consistent with immunohistochemistry investigations (Supplementary Figures S5, S6), Western blot showed that collagen I was significantly increased in LA (by 76%), SAN (by 76%), AVJ (by 65%), LPF (by 46%), and RV (by 55%) (Figure 6C). Cav3 was significantly decreased in the AVJ (by 26%) and LPF (by 32%) (Figure 6D). Figure 6E shows specific Western blot bands of collagen I and Cav3 in the different regions of the heart.

Apoptosis was investigated using TUNEL staining (green nuclei signal in Supplementary Figure S3). Cav3 was also immunolabeled (red signal at the cell membrane of the cardiac cells in Supplementary Figure S3). TUNEL labels the ends of DNA strand breaks characteristic of the apoptotic process. Supplementary Figure S3 shows TUNEL-positive regions, including the RA, LA, and RV, in the STZ rat heart unlike control heart. The cells of the CCS were all negative for TUNEL staining.

## Discussion

### Reduced Membrane Clock Protein Expression in STZ Rat Heart

This study has shown downregulation of key ion channels proteins important for healthy functioning of the CCS. Previous research has shown significant downregulation of HCN4 in the SAN of STZ rat heart (Huang et al., 2017). Our study has also demonstrated that HCN4 is downregulated in the SAN in the STZ rat compared to controls. Our experiments also demonstrated that blocking of HCN channels with CsCl slowed the beating rate of rat SAN preparations, which indicates that HCN channels play a critical role in maintaining spontaneous pacemaking (Dobrzynski et al., 2013). Consistent with the downregulation of HCN4, *I*_f_ in the SAN was reduced in STZ rat which might explain the reduced SAN function. The downregulation of HCN4 and *I*_f_ might contribute to bradyarrhythmia in human TIDM.

Ca_v_1.3 is one of the Ca^2+^ channels responsible for *I*_CaL_ (Christel et al., 2012). Ca_v_1.3 has been shown to play an important role in *I*_CaL_ in the nodal cells of SAN and AVJ (Marger et al., 2011). Mice with deletion of Ca_v_1.3^–/–^ show a prolonged PR interval and complete AV block (Striessnig et al., 1998). Previous studies have shown reduced cardiac contractility and *I*_CaL_ amplitude and density in the ventricle of STZ mouse heart (Bracken et al., 2006). Our study is the first to show a significant downregulation of Ca_v_1.3 protein in the SAN and AVJ in the STZ rat. Downregulation of Ca_v_1.3 could contribute to bradyarrhythmia and heart block in human TIDM. Downregulation of Ca_v_1.3 could also contribute to bradyarrhythmia and heart block in human TIDM.

T-type Ca^2+^ channels (mainly Ca_v_3.1) are known to be highly expressed in the CCS (Chandler et al., 2009) where it contributes to the generation of the pacemaker potential (Bohn et al., 2000). It has previously been reported that mice with deletion of Ca_v_3.1^–/–^ show significantly decreased intrinsic (*in vivo)* heart rate, prolonged SAN recovery time and decreased pacemaker activity in isolated SAN cells (Hansen, 2015). Our study is the first to show downregulation of Ca_v_3.1 in the SAN at the protein level in the STZ rat.

Collectively, downregulation of HCN4, Ca_v_1.3 and Ca_v_3.1 (Table 2) could explain the prolonged RR and PR intervals observed in the *ex vivo* ECG in our study (Table 1).

**TABLE 2:** Summary of altered proteins in STZ-induced type I diabetic hearts comparing to control: variable proteins investigated between STZ-induced diabetic rats and age-matched controls.

**Protein investigated**	**LA**	**SAN**	**AVJ**	**LPF**	**RV**
HCN4		↓			
Na_v_1.5	↓				↓
Ca_v_1.3		↓			
Ca_v_3.1		↓			
RyR2	↓		↓	↓	↓
NCX1	↓	↓	↓	↓	↓
α-actinin				↓	
Cx40				↓	
Cx43	↓			↓	↓
Cx45		↓		↓	↓
NF-M	↓	↓	↓	↓	↓
β_2_-AR		↓	↑		
Collagen I	↑	↑	↑	↑	↑
Cav3			↓	↓	

*↓ indicates significant decrease from control data (*P* < 0.05); ↑, significant increase from control data (*P* < 0.05). LA, left atrium; SAN, sinoatrial node; AVJ, atrioventricular junction; LPF, left Purkinje fiber; RV, right ventricle; Cav3, Caveolin 3; β_2_-AR, β_2_ adrenergic receptor.*

Na_v_1.5 is involved in the initiation and conduction of action potentials in the heart. Heterozygous knockout mice Na_v_1.5^+/-^, show slower conduction ability in the whole heart and significantly increased risk of ventricular arrhythmia (Royer et al., 2005). Recent studies have demonstrated that the density of *I*_Na_ is significantly reduced in the ventricle of diabetic rabbits as compared to controls and the protein level of Na_v_1.5 tends to be lower in diabetic hearts (Stables et al., 2014). Consistent with previous research, our study also shows significant downregulation of Na_v_1.5 in the working myocytes. This downregulation could be a factor responsible for ventricular arrhythmogenesis in diabetes (Stables et al., 2014). Downregulation of Na_v_1.5 protein in T1DM might also be a factor responsible for the QRS complex prolongation (Table 1).

### Reduced Calcium Clock Protein Expression in STZ Rat Heart

Our study shows significant downregulation of Ca^2+^- handling proteins in the CCS and working myocardium. It has been previously observed that the threshold for activation of RyR2 by luminal Ca^2+^ binding is reduced in type I diabetic cardiomyocytes (Yaras et al., 2005). Studies have shown that NCX1 mRNA and protein expression in the ventricles are significantly downregulated in STZ-induced T1DM (Zhong et al., 2001). In agreement with previous studies, our study shows significant protein downregulation of RyR2 and NCX1 in the working myocytes, which is expected to cause contractile dysfunction in T1DM in humans. Ca^2+^-handling proteins are involved in the Ca^2+^ clock pacemaker mechanism (Monfredi et al., 2013) and downregulation of RyR2. NCX1 within the CCS in the STZ rat could be involved in the prolongation of RR and PR intervals as well as QRS complex in the diabetic heart (Table 1).

### Reduced Gap Junctional Proteins in STZ Rat Heat

It is well known that the gap junction plays a vital role in electrical cell-to-cell coupling and impulse propagation between myocytes. The electrical resistance of the gap junction is much lower than that of the surface membrane (Boyett et al., 2006). Research involving humans (Yang et al., 1990) and animal models (Howarth et al., 2008) of diabetes show a prolonged QRS complex, indicating impaired conduction of the electrical impulse in the His-Purkinje system. There is no previous investigation of Cx40 protein in the PF in an experimental model of diabetes. This study is the first to demonstrate that Cx40 is significantly downregulated in the LPF in the STZ rat. The decrease in expression of Cx40 in the PFs in diabetes is expected to slow PF conduction and help explain the QRS complex prolongation (Table 1). Changes in the gap junction distribution are a common characteristic of different pathologies. Results obtained by co-labeling Cx40 with Cav3 show redistribution of Cx40 from the intercalated disc to the peripheral side membrane of the PF cells in the STZ rat (Supplementary Figure S4). The lateralization of Cx40 could affect conduction in PFs in the diabetic heart.

A previous animal study reported decreased intercellular communication with high glucose levels and demonstrated that Cx43 mRNA and protein in diabetic ventricular muscle is decreased (Howarth et al., 2008). In line with these findings, our study has demonstrated a significant downregulation of Cx43 protein in the atrium, PFs and ventricles. These changes might also help explain the QRS complex prolongation (Table 1).

In a previous study involving STZ rats, altered Cx45 mRNA expression in the SAN and ventricular myocytes was observed (Severs et al., 2004). Our study has demonstrated a significant downregulation of Cx45 protein in the SAN, LPF, and RV. This downregulation of Cx45 in the SAN and PFs helps explain the RR interval and QRS complex prolongation in T1DM (Table 1).

### Reduced Neuronal Control of Heart Rhythm in STZ Rat

A previous study reported that the expression of the cytoskeletal NF was downregulated in the hearts in type I diabetic rat (Sima et al., 2008). Our study has demonstrated downregulation of NF-M in all components of the CCS as well as in the working myocardium in STZ rat heart. Reduced levels of NFs is associated with decreased axonal transport and impaired axonal function (Sayers et al., 2003). Furthermore, reduced β-adrenergic sensitivity has been reported in type I diabetic patients. Our data extends those obtained in previous studies, showing alteration in β_2_-adrenoceptor levels in the CCS in the STZ-induced T1DM. In our study, there was a downregulation of β_2_-adrenergic receptor in the SAN and AVJ. Our data help explain the prolongation of RR, PR, and QT intervals in TIDM, because sympathetic nerve stimulation accelerates heart rate and AVN conduction and shortens the ventricular action potential.

### Apoptosis and Collagen 1 Expression in STZ Rat

Apoptosis, a programmed cell death mechanism, plays a critical role in the pathogenesis of diabetic cardiomyopathy (Korkmaz-Icoz et al., 2015). Cardiac cell death is closely related to compensatory cellular hypertrophy and reparative fibrosis (Hearse and Bolli, 1992). An 85-fold increase in the cardiac myocyte apoptosis was observed in diabetic human heart and an aggregate 30% loss was observed in diabetic rat heart (Fiordaliso et al., 2000). Our study has shown that there is apoptosis in the atria and ventricles in STZ rat but surprisingly not in the CCS. Perhaps the cells of the CCS are less prone to apoptosis.

Furthermore, in line with previous studies in the diabetic ventricle (Black et al., 2010) we also observed increased collagen I expression in the LA and RV. Our study is the first to show a significant increase in the levels of extracellular collagen I expression in all regions of the CCS. Fibrosis has a detrimental impact on cardiac function and contributes to the increased risk of heart failure. T1DM induced fibrosis is a known predictor of cardiac dysfunction and mortality in humans and in experimental animal studies (Furberg, 1999).

### Cellular Hypertrophy and T-Tubules Remodeling in STZ Rat Heart

Hypertrophy was not investigated in this study. Previous studies have reported, cardiac hypertrophy, which were mainly related to the working myocardium (Fiordaliso et al., 2004, 2006; Gurusamy et al., 2005). The focus of this study was to measure the cell capacitance of nodal cells from STZ rat heart compared to controls. This is the first study where we report that cell capacitance decreases in nodal cells in STZ rat.

Altered T-tubules structures in the working myocardium may also contribute to the phenotype of diabetes in STZ rat heart. As T-tubule structures are complex and generally not well developed in the CCS, this study does not provide any hypothetical explanations on these complex structures. In fact, T-tubules remodeling in STZ rat in the working myocardium has been previously investigated and reported. In brief, there is prolongation of action potential duration, slower Ca^2+^ transient decay, reduced myofilament Ca^2+^ sensitivity, decreased regular T-tubules in appearance, and increased extracellular collagen I (Ward and Crossman, 2014). Evidence supporting significant remodeling of T-tubules in type 2 diabetes mellitus has also been reviewed previously (Russell et al., 2017).

### Potential Interventions

For potential interventions to rescue key ion channels expression suppression of miR-423-5p can be carried out as it has been shown that this microRNA reversed training-induced bradycardia via upregulation of HCN4 and *I*_f_ (D’Souza et al., 2017). There are many other approaches that can be considered. Treatment with bisoprolol has been shown to partially reverse both the SAN function and HCN4 mRNA expression (Du et al., 2016). GDF-15 (growth/differentiation factor 15) has been shown to enhance protein expression of Ca_v_1.3 via TRII in rat (Lu et al., 2016). Clarin-1 gene transfer has been shown to upregulate Ca_v_1.3 expression and activity (Dulon et al., 2018). Aβ_25__–__35_ could also chronically enhance expression Ca_v_1.3 in the rat (Kim and Rhim, 2011). Cdk5 has been shown to stably expressing Ca_v_3.1 channel and upregulate macroscopic currents in HEK-293 cells (Calderon-Rivera et al., 2015). Aldosterone has been shown to stimulate miR-204 and further promotes the expression of Ca_v_3.1channels in isolated rat ventricular cardiomyocytes (Koyama et al., 2018). Additionally, MOG1 has been shown to enhance Na_v_1.5 expression and serve as a therapeutic target for sodium channelopathies (Chakrabarti et al., 2013). FoxO1 has been also shown to promote of Na_v_1.5 expression in the heart (Cai et al., 2014).

In order to rescue RyR2 and NCX1 expression in STZ rat hearts, PKP2 has been shown to promote RyR2 expression (Cerrone et al., 2017),miR-106b-25 has been shown to enhance RyR2 expression (Chiang et al., 2014). Dexrazoxane has also been proven to prevent the decrease in RyR2 mRNA in daunorubicin-treated rats (Burke et al., 2000). Anti-miR-214 could be used to rescue decreased NCX1 proteins (Aurora et al., 2012). KB-R7943 has been shown to enhance NCX1 expression via chronically β-Adrenergic receptor-stimulation in the heart (Xu et al., 2009).

Gap junctional proteins have been shown to be regulated by many transcription factors, including Sp1, Sp3, and AP1 (Teunissen and Bierhuizen, 2004). Others including thyroid, estrogens and signaling via Ras, Wnt1, and cAMP pathways may also affect gap junctional proteins expression (Leybaert et al., 2017). Histone acetylation has been shown to stimulate the expression of Cx43 and Cx45, as induced by chemical inhibitors of histone deacetylase (HDAC) enzymes (Hattori et al., 2007). miR-1, miR-17-92, and miR-130a have been shown to exacerbates arrhythmogenesis by reducing Cx40 and Cx43 expression (Yang et al., 2007; Danielson et al., 2013; Osbourne et al., 2014), anti-miR could be used to rescue decreased gap junctional proteins. Additionally, miR-208a has been shown to promote Cx40 expression (Callis et al., 2009).

There are a few potential interventions to be considered to rescue neuronal control of heart rhythm, collagen I expression and apoptosis in STZ induced diabetic hearts. Indacaterol and corticosteroids could be considered to reverse the function and expression of β_2_-adrenergic receptors (Rinaldi et al., 2015; Scott et al., 2016). Cryptic amyloidogenic elements and RE1 silencing transcription factor has been shown to regulate the expression of NFs (Ching and Liem, 2009; Rebelo et al., 2016). Furthermore, miR-223 has been shown to inhibit collagen I expression via RASA1 in order to prevent cardiac functional deterioration and cardiac fibrosis (Liu X. et al., 2018). TRPV3 could also be inhibited to downregulate the expression of collagen I against cardiac fibrosis (Liu Y. et al., 2018). In addition, physical high-intensity resistance training might also help in managing collagen accumulation in the diabetic heart (Guzzoni et al., 2017). In order to rescue myocardial apoptosis in STZ rat hearts, (Pro)renin receptor has been shown to inhibit apoptosis, and inflammatory response in rats with diabetic cardiomyopathy via extracellular signal-regulated kinase/ROS pathway (Dong et al., 2019). Decoy receptor-3 has been recently shown to regulate apoptosis via PI3K/AKT signaling pathway (Chen et al., 2019).

## Conclusion

Dysfunction of the CCS plays a critical role in a variety of cardiac arrhythmias (Dobrzynski et al., 2013). In STZ T1DM rats, RR interval, PR interval, and QRS complex duration were significantly increased. The beating rate of the isolated SAN was significantly decreased in STZ rats. I_f_ current density and cell capacitance were significantly decreased in STZ rat sinus node cells. Fibrosis but not apoptosis, contributes to dysfunction of the CCS in diabetic rats. As shown in summary Table 2, reduced HCN4, Ca_v_1.3, Ca_v_3.1, NCX1, and Cx45 protein expression in the SAN is expected to contribute to bradyarrhythmia. Reduced RyR2, and NCX1 protein expression in the AVJ is expected to partly contribute to the prolongation of the PR interval. Reduced RyR2, NCX1, Cx40, Cx43, and Cx45 protein expression in the PFs is expected to partly contribute to the prolongation of QRS complex. The downregulation of NF-M and β_2_-adrenergic receptor could be linked to the reduced autonomic control of the heart. Arrhythmias are a major cause of mortality in patients with diabetes (Nakou et al., 2012), it is therefore vital to determine the molecular basis of cardiac electrical dysfunction in T1DM.

## Limitation and Strength

This study focuses on the CCS remodeling. Our study showed for the first time that proteins involved in the function of the CCS were significantly decreased in this model of diabetes (see Table 2 for summary) and these changes can explain observed changes in *ex-vivo* ECG parameters (see Table 1). This is the main strength of our study. The main limitation is some contamination as the components of the CCS are embedded in the working myocardium.

## Ethics Statement

Procedures were carried out in accordance with the regulations of the United Kingdom Animals (Scientific Procedures) Act 1986 and/or United Arab Emirates.

## Author Contributions

YZ carried out the most of experiments, analyzed and illustrated the data, and wrote the manuscript. YW and JY performed the patch clamp and recorded the extracellular potential experiments, respectively. MQ, NG (with help of SK), and FH developed the STZ-induced type I diabetic rat model. SL helped in *ex vivo* functional experiments and edited the manuscript. HS reviewed the manuscript and partly supervised YZ. MB helped with interpretation of functional experiments and edited the manuscript. HD designed and helped with experiments, supervised YZ, helped with the analysis of data and reviewed the manuscript. All authors reviewed and approved the manuscript.

## Conflict of Interest Statement

The authors declare that the research was conducted in the absence of any commercial or financial relationships that could be construed as a potential conflict of interest.
