# Supplementary material for: Electrical Conduction System Remodeling in Streptozotocin-Induced Diabetes Mellitus Rat Heart
Source: Front Physiol. 2019 Jul 8;10:826. doi: 10.3389/fphys.2019.00826 (PMC6628866; doi:10.3389/fphys.2019.00826)
Supplement: Supplementary file 1 [file Data_Sheet_1.docx]

Supplementary Material

**Electrical conduction system remodelling in streptozotocin-induced diabetes mellitus rat heart**

Yu Zhang^1,3^, Yanwen Wang^1^, Joseph Yanni^1^, Mohammed Anwar Qureshi^2^,

Sunil Jit R.J. Logantha^1^, Sarah Al-Adhamn^1^, Mark R. Boyett^1^, Natalie Gardiner^1^, Hong Sun^3^

Frank Christopher Howarth^2^, Halina Dobrzynski^1*^

*** Correspondence:** Halina Dobrzynski

Email: Halina.Dobrzynski@manchester.ac.uk

**Table S1. Primary antibodies used for immunohistochemistry and western blotting.** Gt, goat; Ms, mouse; Rbt, rabbit; Mono, monoclonal; Poly, polyclonal.

| **Primary antibodies** | **Company** | **Catalogue number** | **Source** | **Dilution** |
| --- | --- | --- | --- | --- |
| Cx40 | Santa-Cruz | SC-20466 | Poly-Gt | 1:100 |
| Cx43 | Millipore | MAB3068 | Mono- Ms | 1:200 |
| Cx45 | Abcam | Ab78408 | Poly- Ms | 1:50 |
| HCN4 | Alomone | APC-052 | Poly-Rbt | 1:50 |
| Ca_v_1.3 | Alomone | ACC-005 | Poly-Rbt | 1:50 |
| Ca_v_3.1 | Alomone | ACC-021 | Poly-Rbt | 1:50 |
| Na_v_1.5 | Alomone | ASC-005 | Poly-Rbt | 1:50 |
| RyR2 | Thermo F | MA3-916 | Mono- Ms | 1:100 |
| NCX1 | Thermo F | C2C12 | Mono- Ms | 1:50 |
| NF-M | Sigma | G9670 | Mono- Ms | 1:100 |
| β_2_-Adrenergic receptor | Abcam | Ab182136 | Mono- Rbt | 1:50 |
| Collagen I | Abcam | Ab34710 | Poly-Rbt | 1:100 |
| Cavolin3 | Transduction | 610421 | Poly-Ms | 1:200 |
| β-Actin | Sigma | A5441 | Mono-Ms | 1:200 |
| α-Actinin | Sigma | Ab18061 | Mono-Ms | 1:100 |

**Table S2. Secondary antibodies used for western blotting.** HRP, horseradish peroxidase conjugated.

| **Secondary antibody** | **Company** | **Catalogue number** | **Dilution** |
| --- | --- | --- | --- |
| Anti-Rbt- HRP | Jackson Lab | 111-035-114 | 1:2000 |
| Anti-Ms- HRP | Life Science | 31460 | 1:1000 |
| Anti-Gt- HRP | Life Science | 31430 | 1:1000 |

**Table S3. Secondary antibodies used for immunohistochemistry.** FITC, fluorescein isothiocyanate; Cy3, cyanine.

| **Secondary antibody** | **Company** | **Cat number** | **Dilution** |
| --- | --- | --- | --- |
| Anti-Rbt-FITC | Millipore | AP132F | 1:400 |
| Anti-Rbt-Cy3 | Millipore | AP132C | 1:100 |
| Anti-Ms-FITC | Millipore | AP124F | 1:400 |
| Anti-Ms-Cy3 | Millipore | AP124C | 1:400 |
| Anti-Gt-Cy3 | Santa Cruz | 166894 | 1:100 |
| TUNEL Kit | Roche | 11 684 795 910 | N/A |

**Table S4. Functional experimental results comparing STZ-induced type I diabetic rats and age-matched control rats. Data are shown in Mean ± SEM, *P<0.05.**

| **SAN beating rate** | **Control** | **STZ** |
| --- | --- | --- |
| **Resting (Before)** | 305.10 ± 11.28 | 252.22 ± 11.12* |
| **After application of 2 mM CsCl** | 221.65 ± 17.21 | 170.66 ± 16.34* |
| **After application of 2 μM ryanodine** | 231.48 ± 16.37 | 160.01 ± 30.23* |
| **Cell capacitance (pF)** | 20.21 ± 1.00 | 14.68 ± 0.93* |
| ***I*_f_ density at -50 mV (pA/pF)** | -0.54 ± 0.26 | -0.48 ± 0.23* |
| ***I*_f_ density at -65 mV (pA/pF)** | -3.21 ± 0.52 | -1.66 ± 0.21* |
| ***I*_f_ density at -80 mV (pA/pF)** | -6.77 ± 1.02 | -4.03 ± 0.38* |
| ***I*_f_ density at -95 mV (pA/pF)** | -11.46 ± 1.62 | -6.99 ± 0.61* |
| ***I*_f_ density at -110 mV (pA/pF)** | -16.18 ± 2.20 | -10.39 ± 0.88* |
| ***I*_f_ density at -125 mV (pA/pF)** | -21.04 ± 2.83 | -14.28 ± 1.18* |


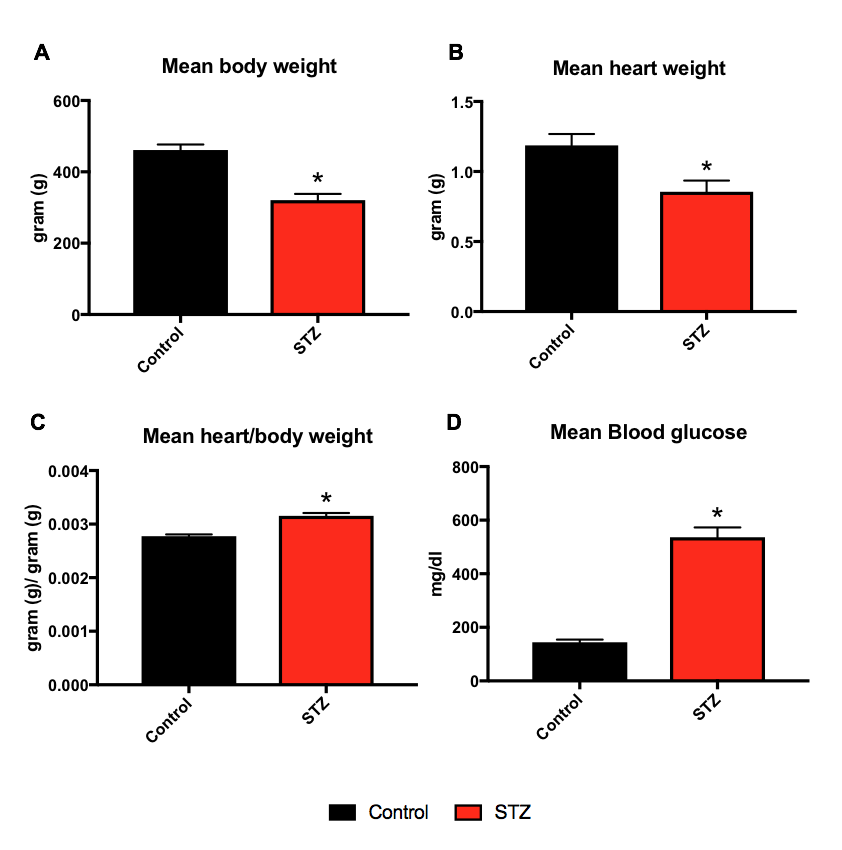


**Figure S1. General characteristics of control and STZ-induced diabetic rats.** (A) Body weight. (B) Heart weight. (C) Heart weight to body weight ratio. (D) Blood glucose. Means ± SEM shown; n= 22 control rats and 21 STZ rats. *P<0.05.


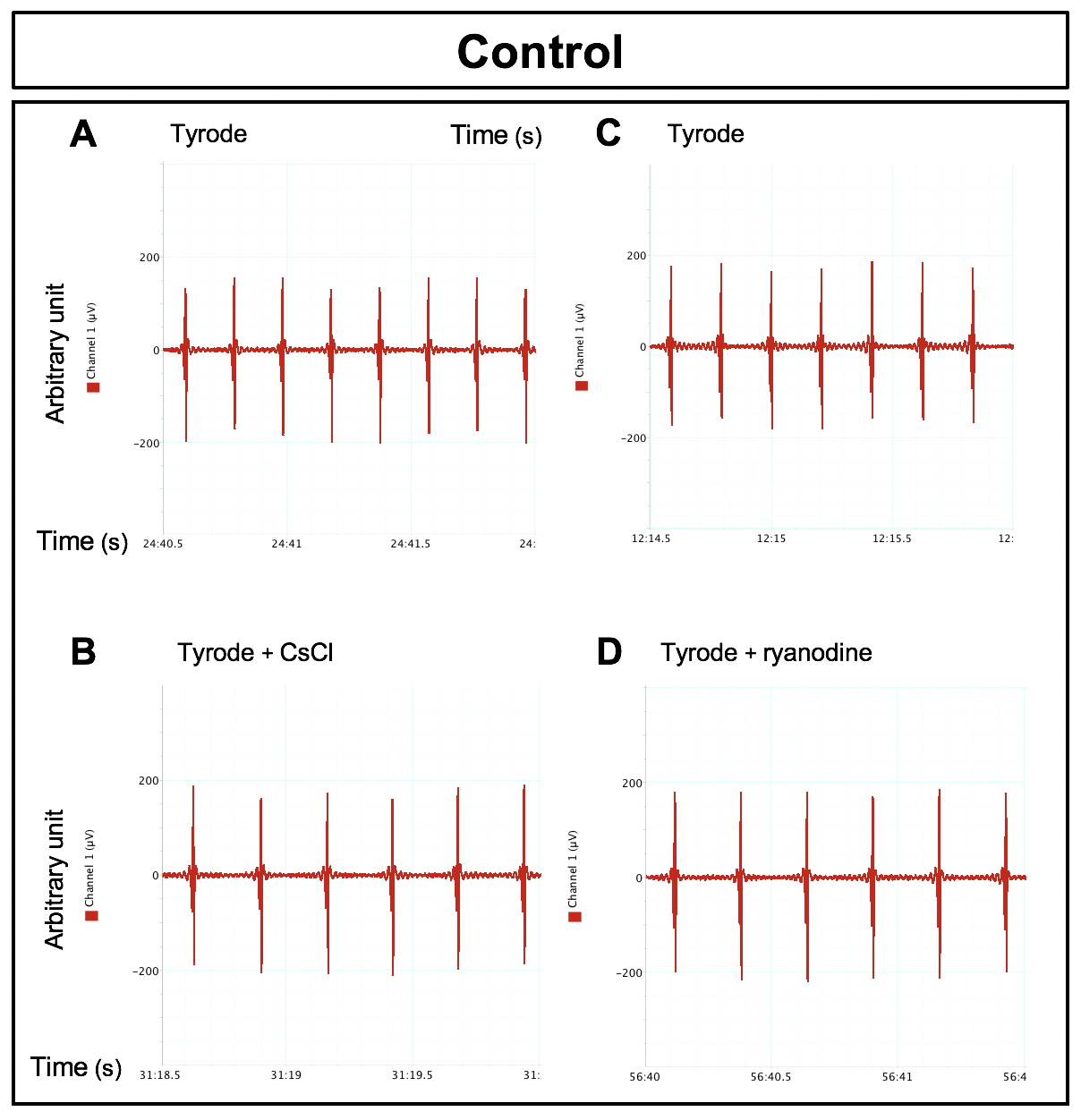


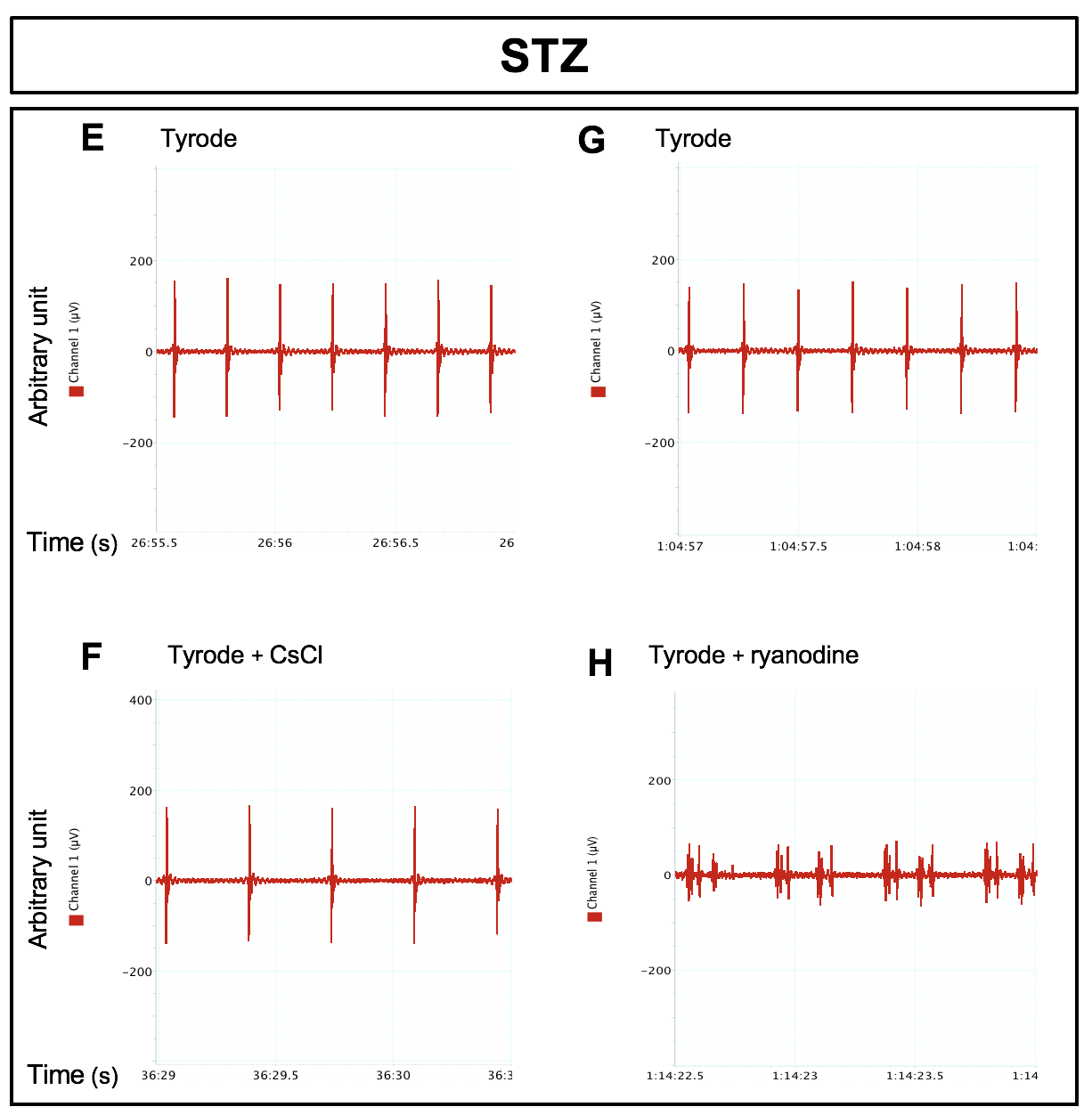


**Figure S2. Effect of Cs^+^ and ryanodine on the rate of beating of the isolated SAN in STZ-induced diabetic rats:** (A-D) Extracellular potential recordings of isolated control SAN, (E-H) Extracellular potential recordings of isolated STZ induced diabetic SAN. Extracellular potential recordings before (A, E) after (B, F) applying 2 mM CsCl. Recordings before (C, G) and after (D, H) applying 2 μM ryanodine.


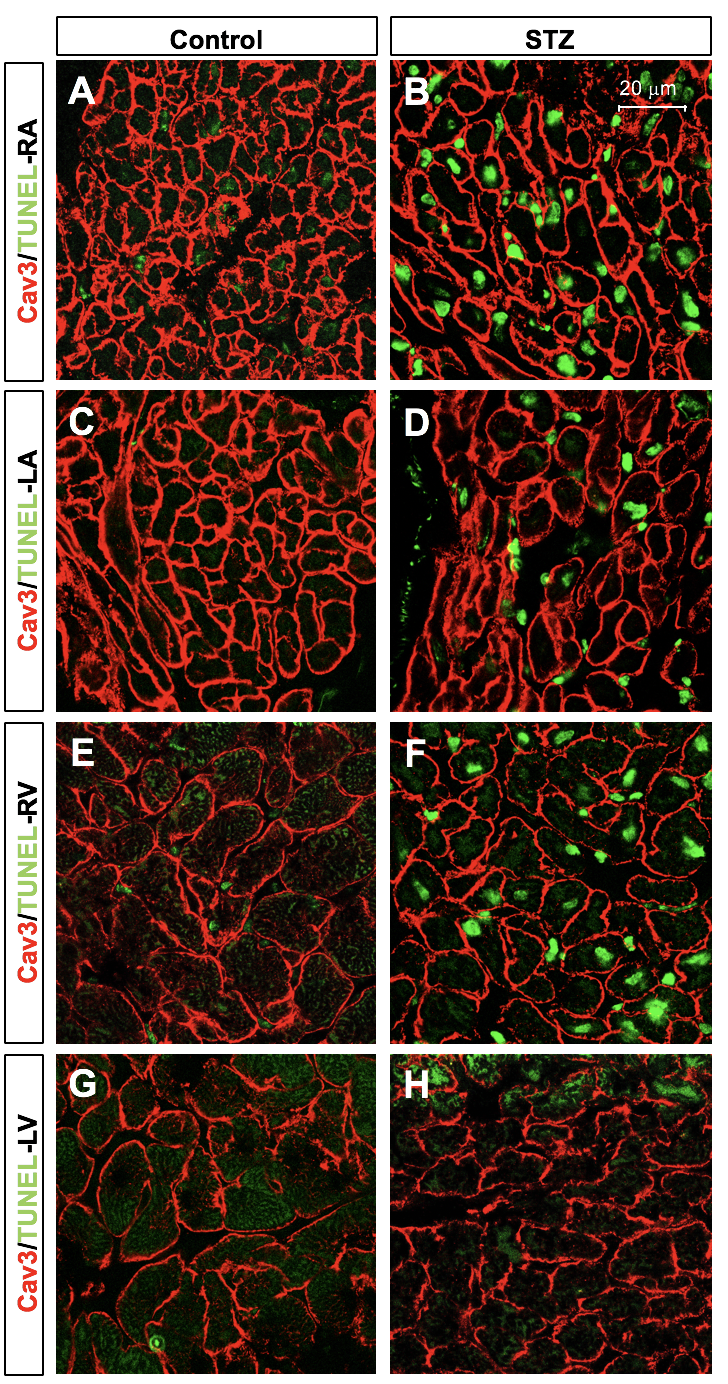


**Figure S3: TUNEL staining of RA, LA, RV and LV of control and STZ induced-type I diabetic rats.** High-power confocal micrographs of the right atrium (RA), left atrium (LA), right ventricle (RV) and left ventricle (LV) labelled for Cav3 (red signal; n=5 for each group) and TUNEL (green signal; n=5 for each group), Bar = 20 μm.


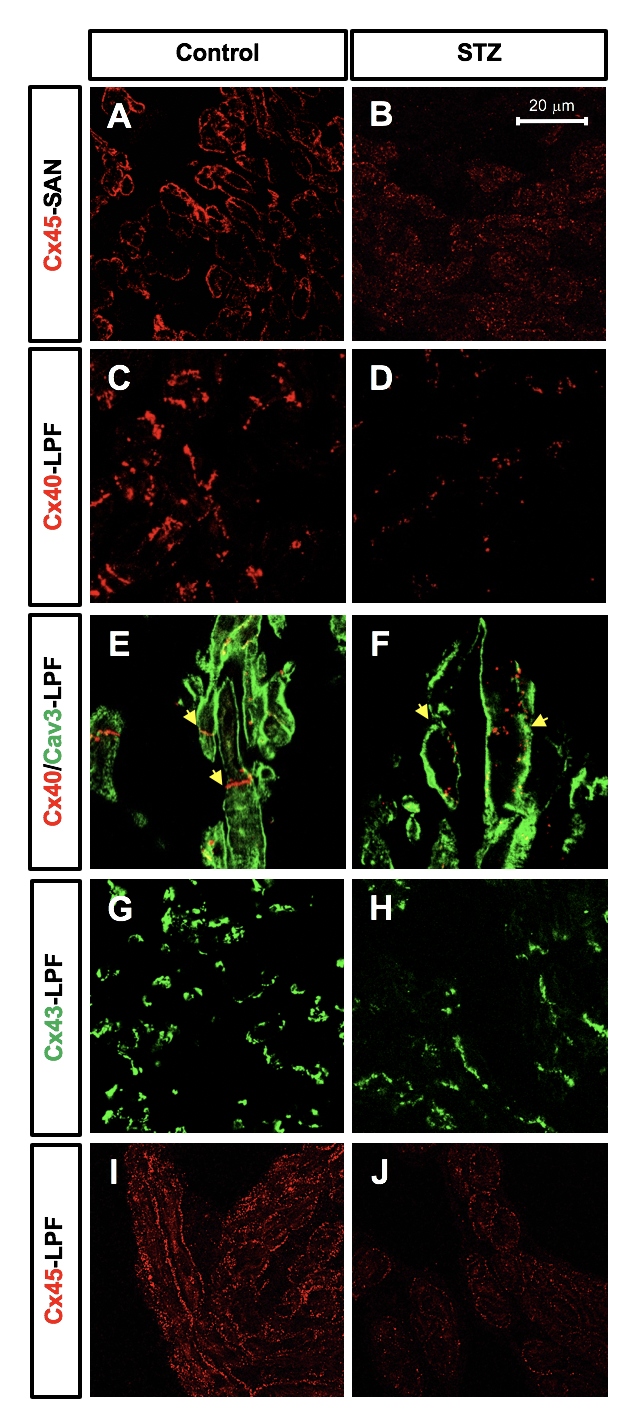


**Figure S4. Cx40, Cx43 and Cx45 distribution in SAN and LPF of the heart in STZ-induced diabetic and control rats: (A-J)** High-power confocal micrographs of the sinoatrial node (SAN) and left Purkinje fiber (LPF), labelled for Cx40 (red signal; n=5 for each group), Cx43 (green signal; n=5 for each group), Cx45 (red signal; n=5 for each group) and Cav3 (green signal; n=5 for each group). LPF, left Purkinje fibers; SAN, sinus node; LPF. Bar = 20 μm.

**
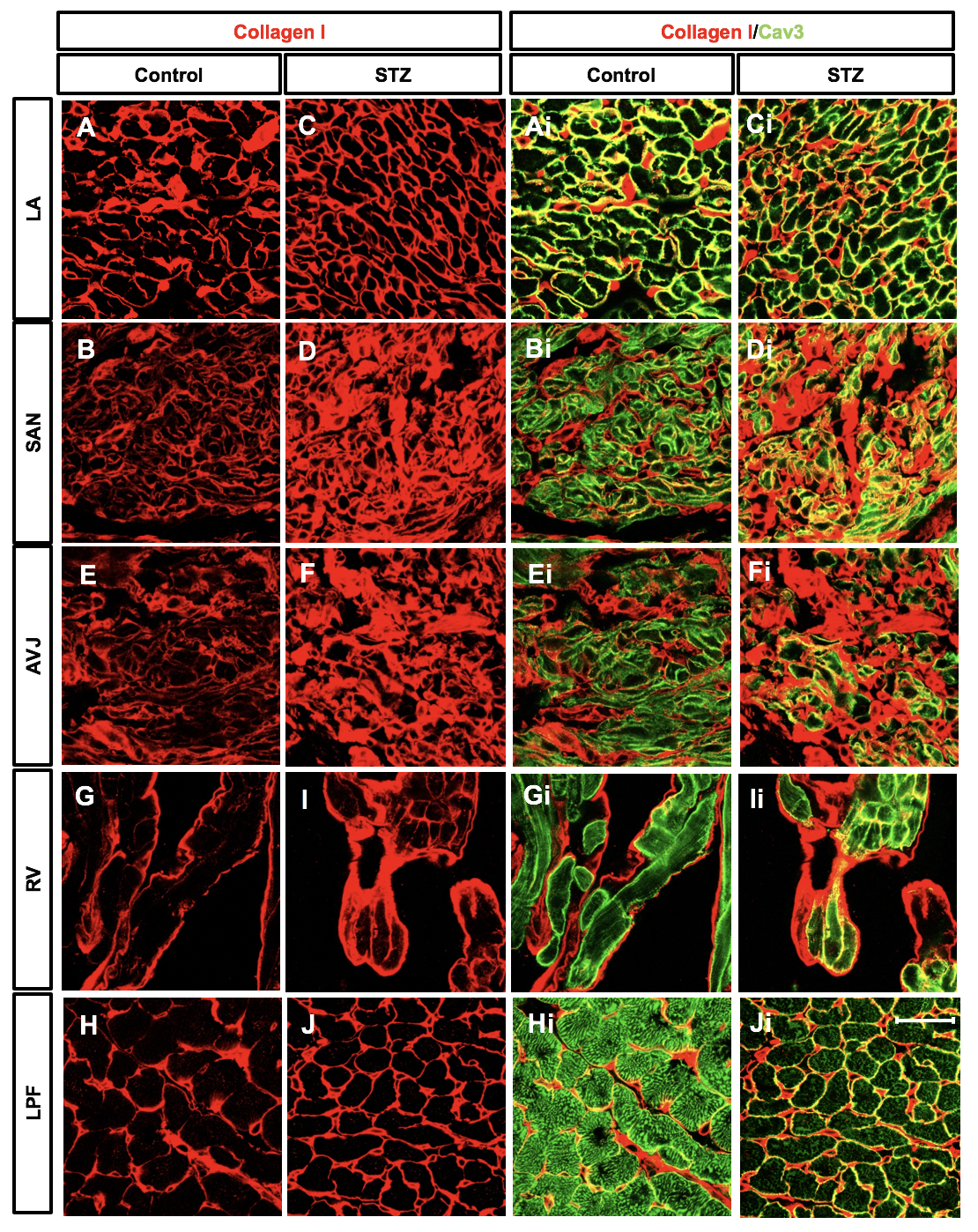
**

**Figure S5. Immunolabelling of collagen I expression in LA, SAN, AVJ, LPF and RV in STZ-induced diabetic rats versus control rats:** High-power confocal micrographs of the LA (left atrium), SAN (sinoatrial node), AVJ (atrioventricular junction), LPF (left Purkinje fibres) and RV (right ventricle), double labelled for collagen I (red signal; n=5 for each group)/Cav3(green signal; n=5 for each group). Bar = 20 μm.

**Figure S6. Semi-quantifications of Collagen I immunohistochemistry in STZ-induced diabetic rats versus control rats**: Mean relative abundance (normalized to β-actin) of collagen I in the LA (left atrium), SAN (sinoatrial node), AVJ (atrioventricular junction), LPF (left Purkinje fibres) and RV (right ventricle). *Significant different from control. Data are mean ± SEM (n = 4 for each group); P<0.05.


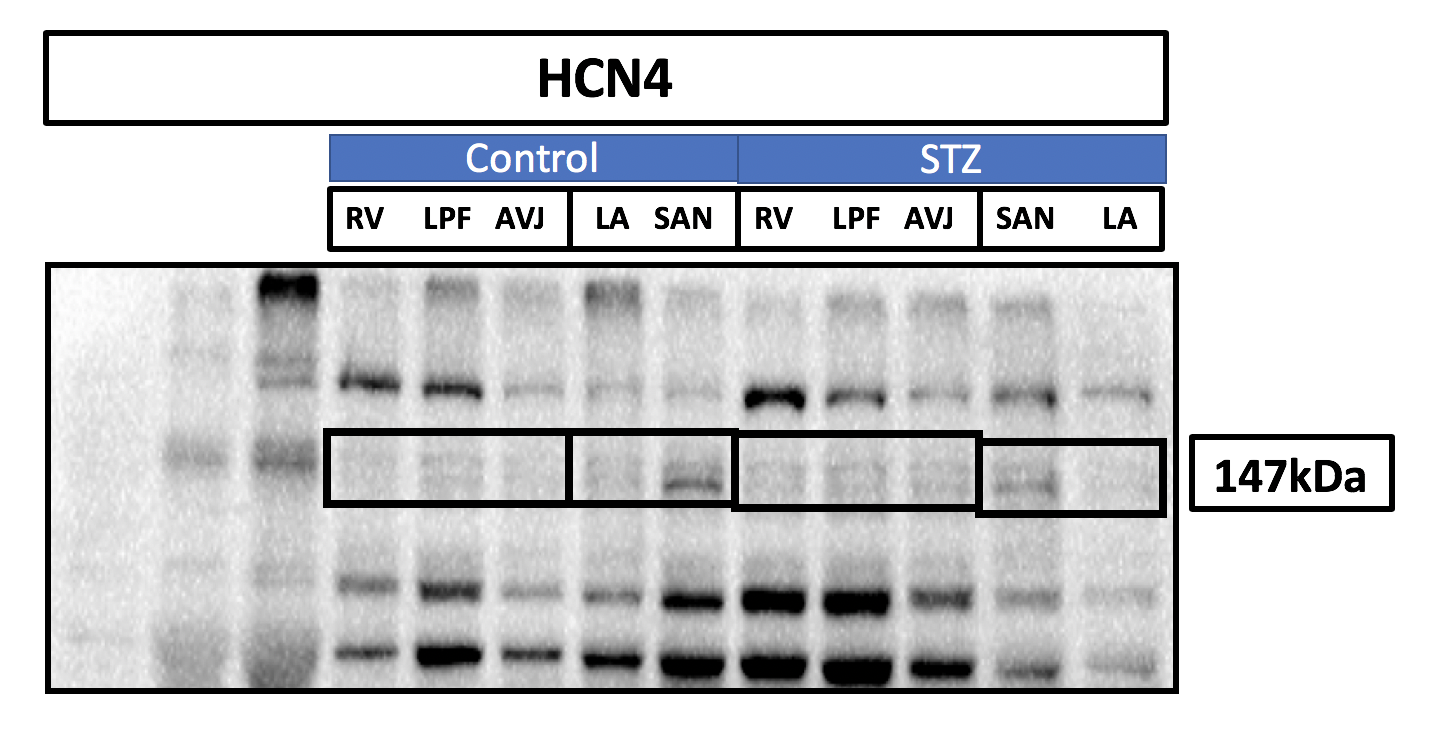


**Figure S7**: **Western blot membrane images of HCN4 in LA, SAN, AVJ, LPF and LV for control and STZ induced diabetic rats**. Black borders around the images represent regions illustrated in Figure 3E for HCN4 where specific bands for this protein were observed at ~147kDa.


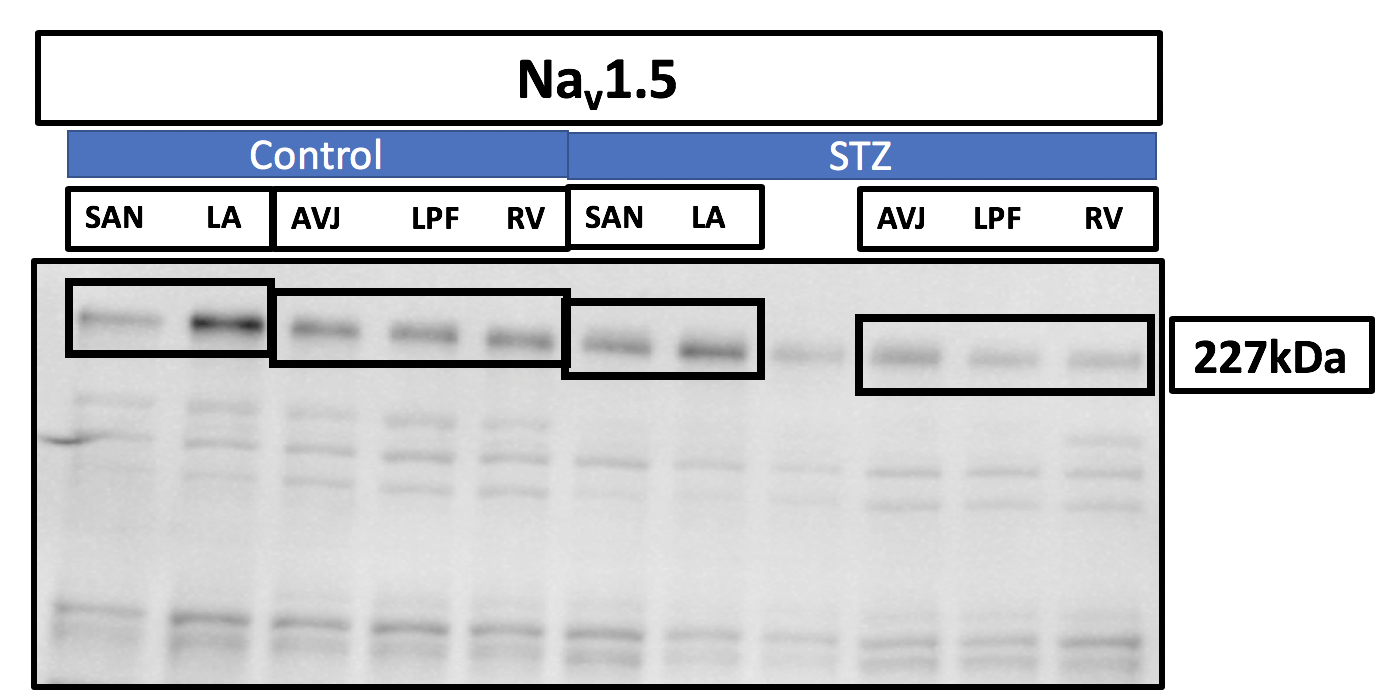


**Figure S8**: **Western blot membrane images of Na_v_1.5 in LA, SAN, AVJ, LPF and LV for control and STZ induced diabetic rats**. Black borders around the images represent regions illustrated in Figure 3E for Na_v_1.5 where specific bands for this protein were observed at ~227kDa.


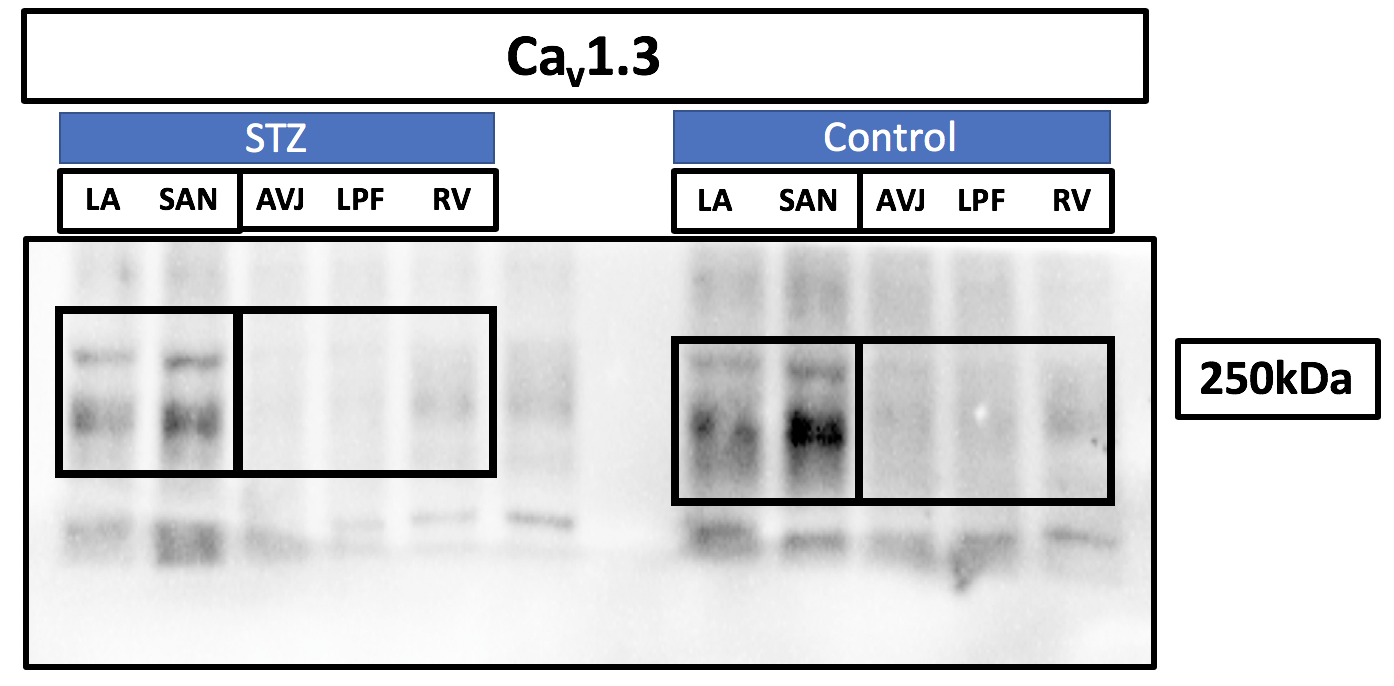


**Figure S9**: **Western blot membrane images of Ca_v_1.3 in LA, SAN, AVJ, LPF and LV for control and STZ induced diabetic rats**. Black borders around the images represent regions illustrated in Figure 3E for Ca_v_1.3 where specific bands for this protein were observed at ~250kDa.


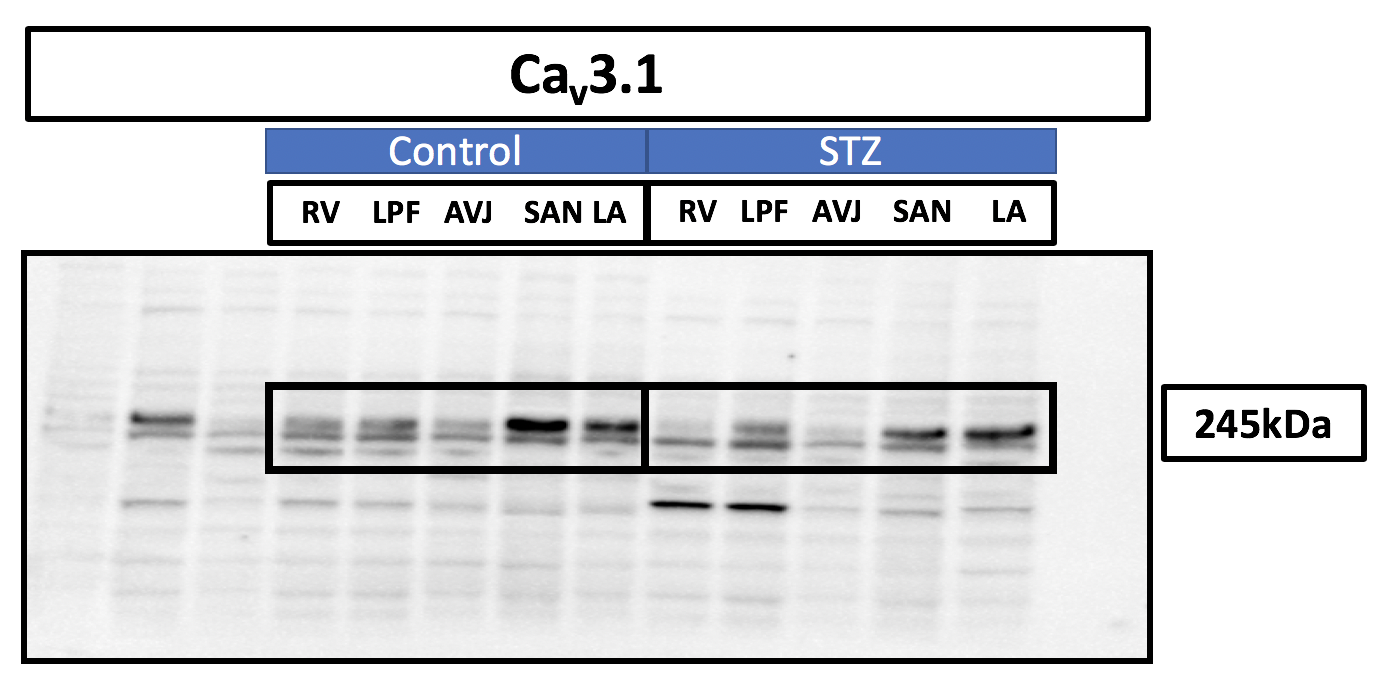


**Figure S10: Western blot membrane images of Ca_v_3.1 in LA, SAN, AVJ, LPF and LV for control and STZ induced diabetic rats**. Black borders around the images represent regions illustrated in Figure 3E for Ca_v_1.3 where specific bands for this protein were observed at ~245kDa.


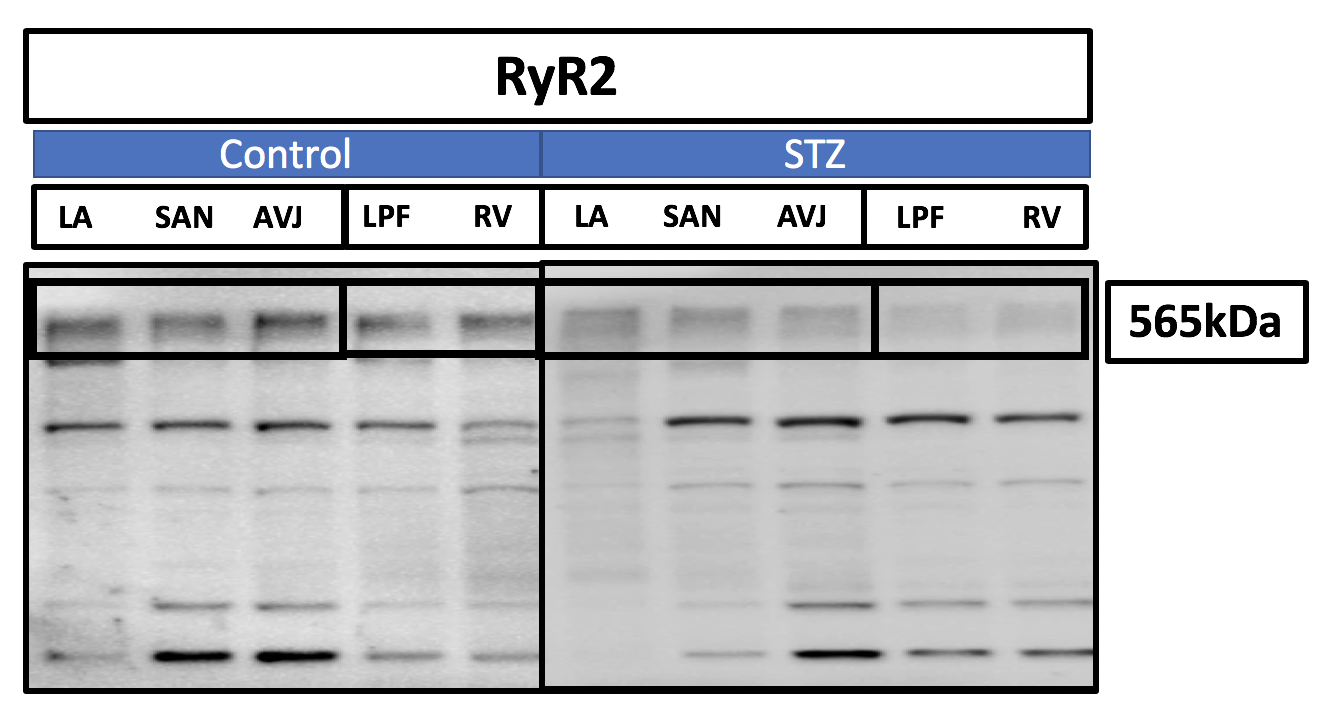


**Figure S11**: **Western blot membrane images of RyR2 in LA, SAN, AVJ, LPF and LV for control and STZ induced diabetic rats**. Black borders around the images represent regions illustrated in Figure 4E for Ca_v_1.3 where specific bands for this protein were observed at ~565kDa.


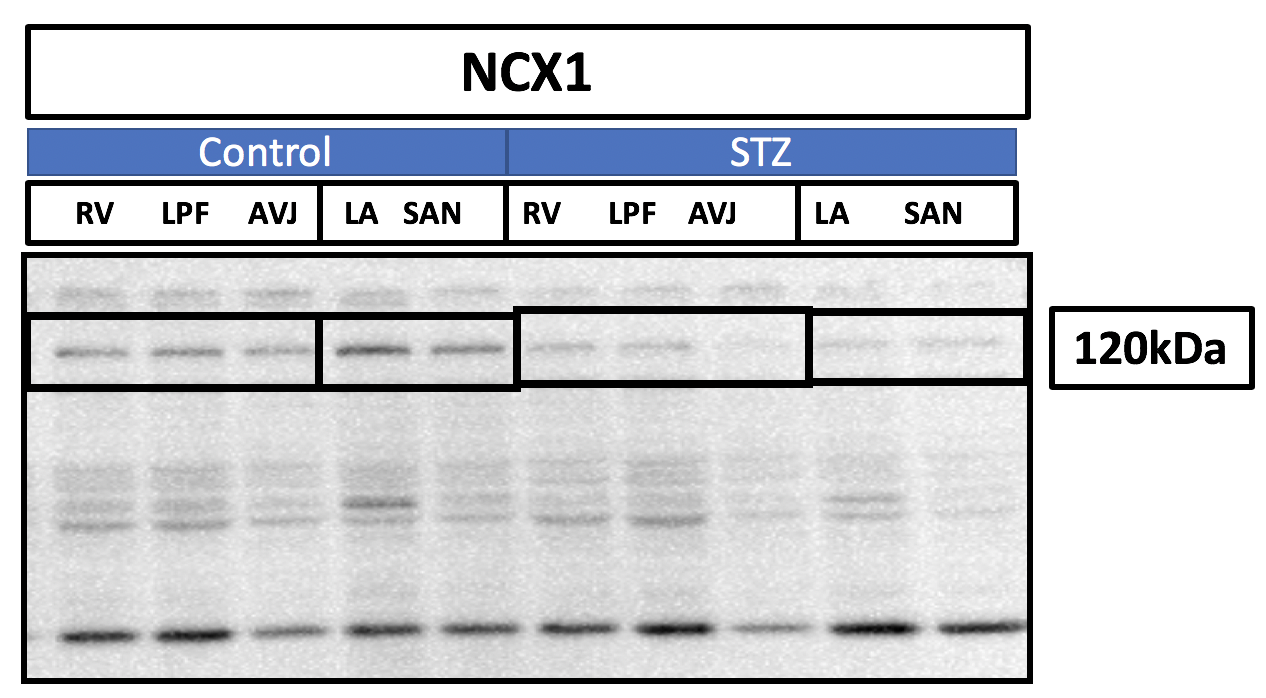


**Figure S12**: **Western blot membrane images of NCX1 in LA, SAN, AVJ, LPF and LV for control and STZ induced diabetic rats**. Black borders around the images represent regions illustrated in Figure 4E for NCX1where specific bands for this protein were observed at ~120kDa.


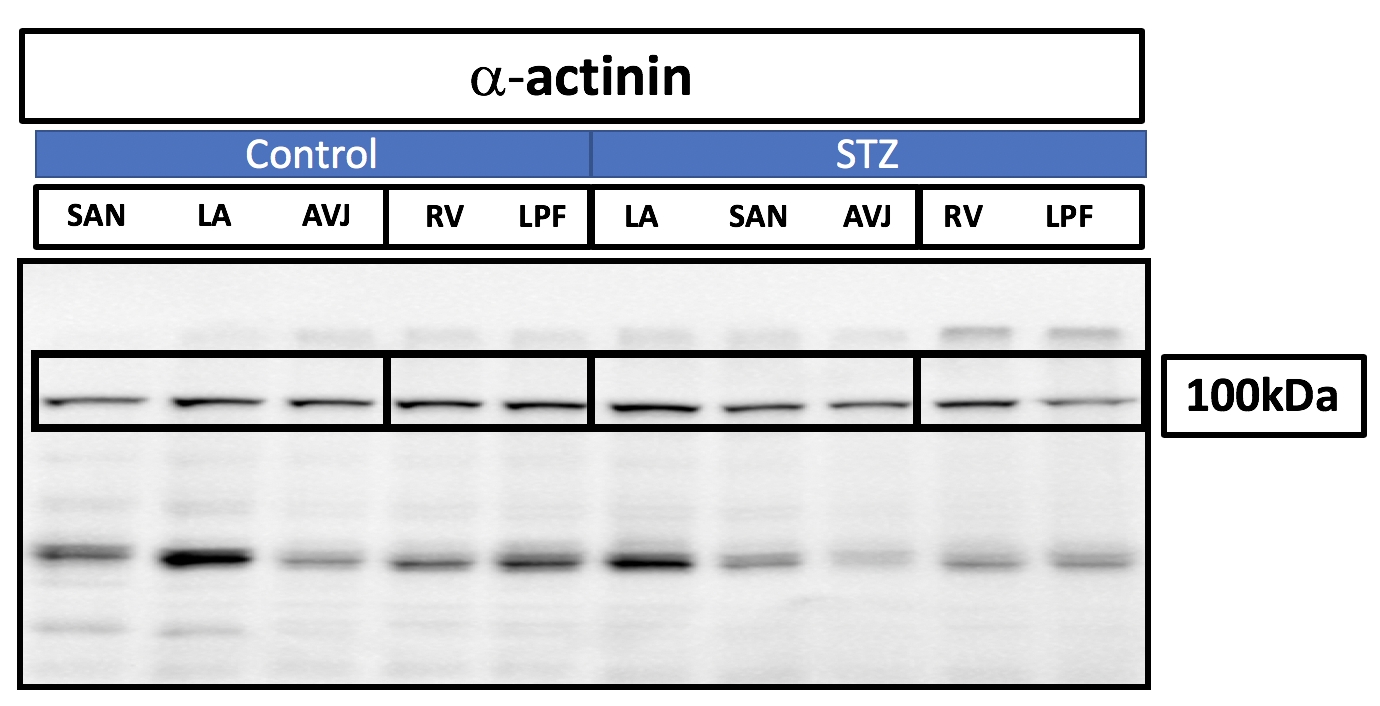


**Figure S13**: **Western blot membrane images of α-actinin in LA, SAN, AVJ, LPF and LV for control and STZ induced diabetic rats**. Black borders around the images represent regions illustrated in Figure 4E for α-actinin where specific bands for this protein were observed at ~100kDa.


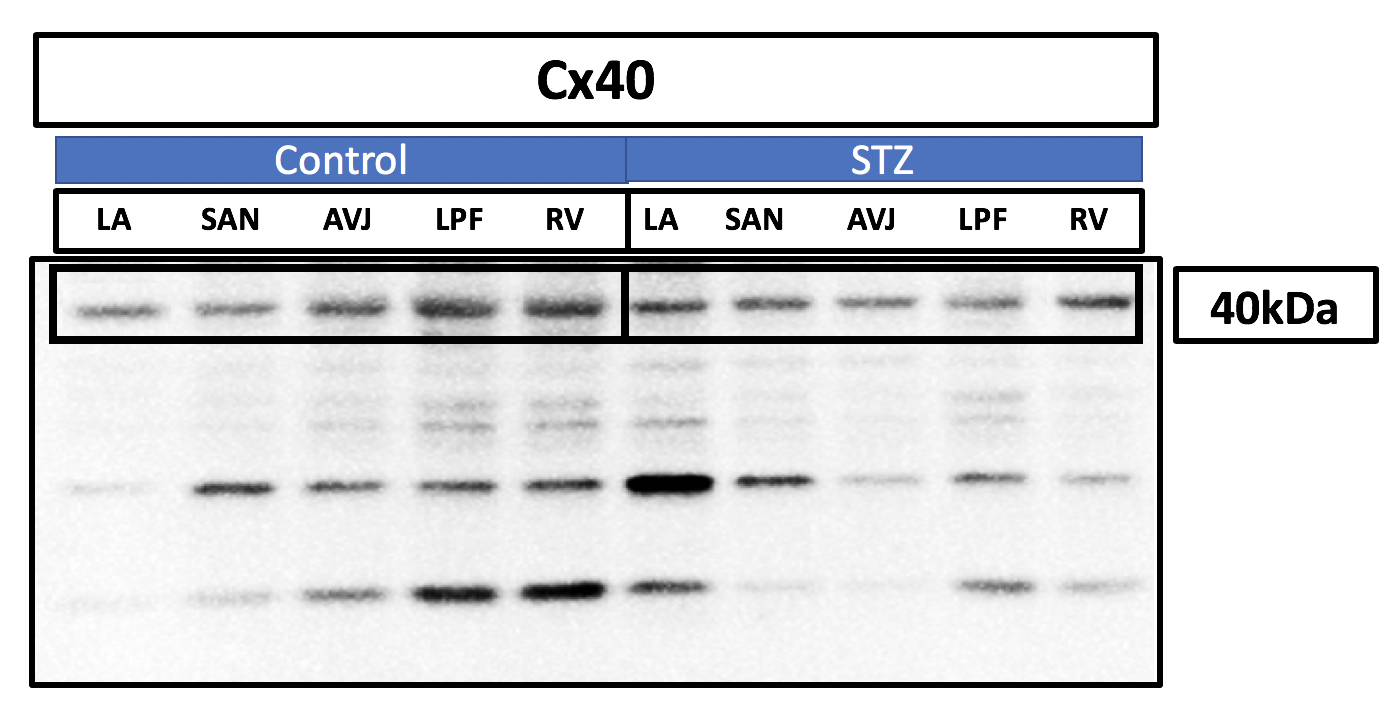


**Figure S14**: **Western blot membrane images of Cx40 in LA, SAN, AVJ, LPF and LV for control and STZ induced diabetic rats**. Black borders around the images represent regions illustrated in Figure 5E for Cx40 where specific bands for this protein were observed at ~40kDa.


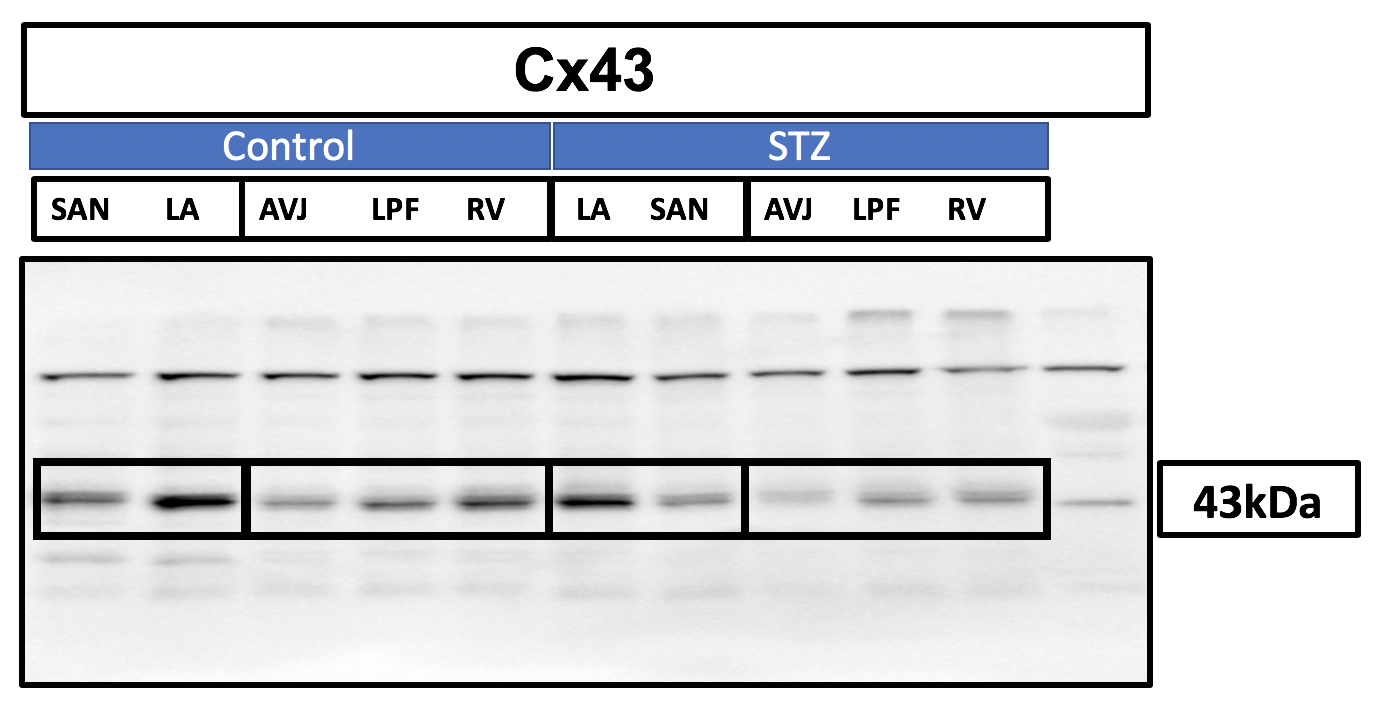


**Figure S15**: **Western blot membrane images of Cx43 in LA, SAN, AVJ, LPF and LV for control and STZ induced diabetic rats**. Black borders around the images represent regions illustrated in Figure 5E for Cx43 where specific bands for this protein were observed at ~43kDa.


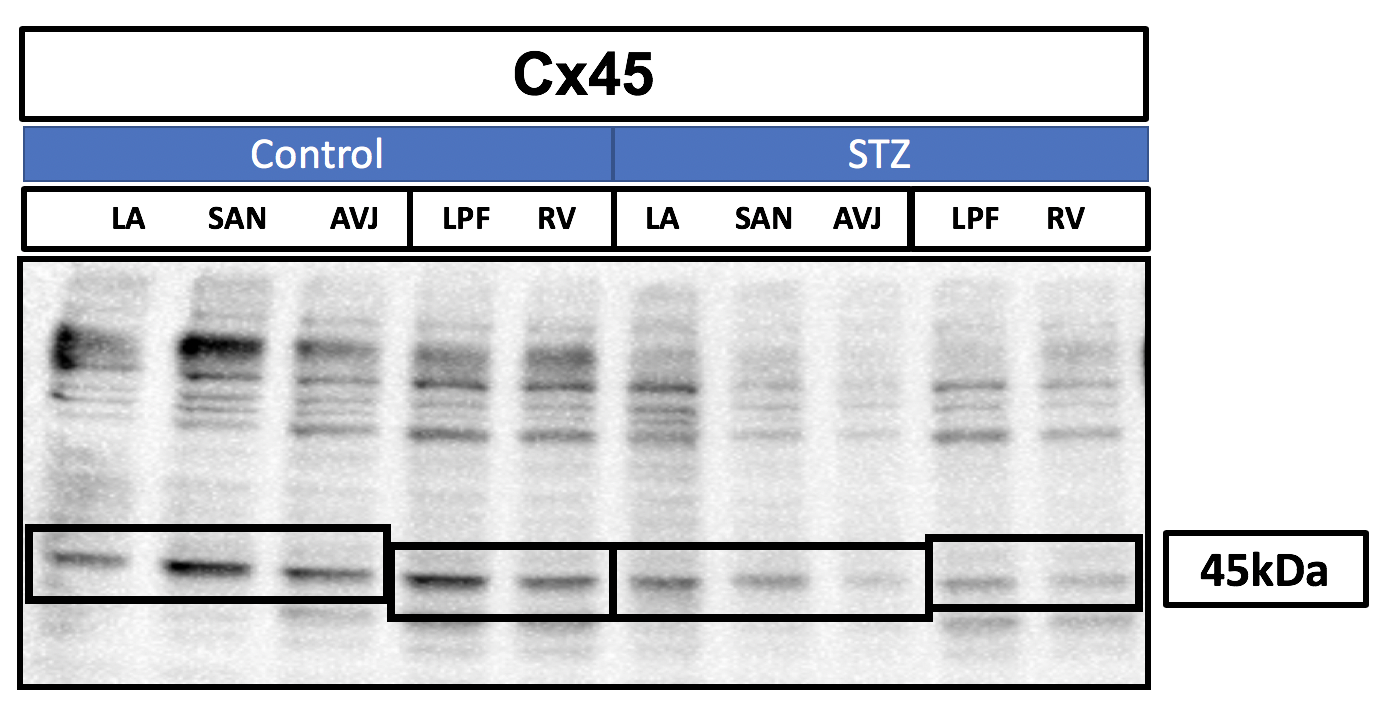


**Figure S16**: **Western blot membrane images of Cx45 in LA, SAN, AVJ, LPF and LV for control and STZ induced diabetic rats**. Black borders around the images represent regions illustrated in Figure 5E for Cx45 where specific bands for this protein were observed at ~45kDa.


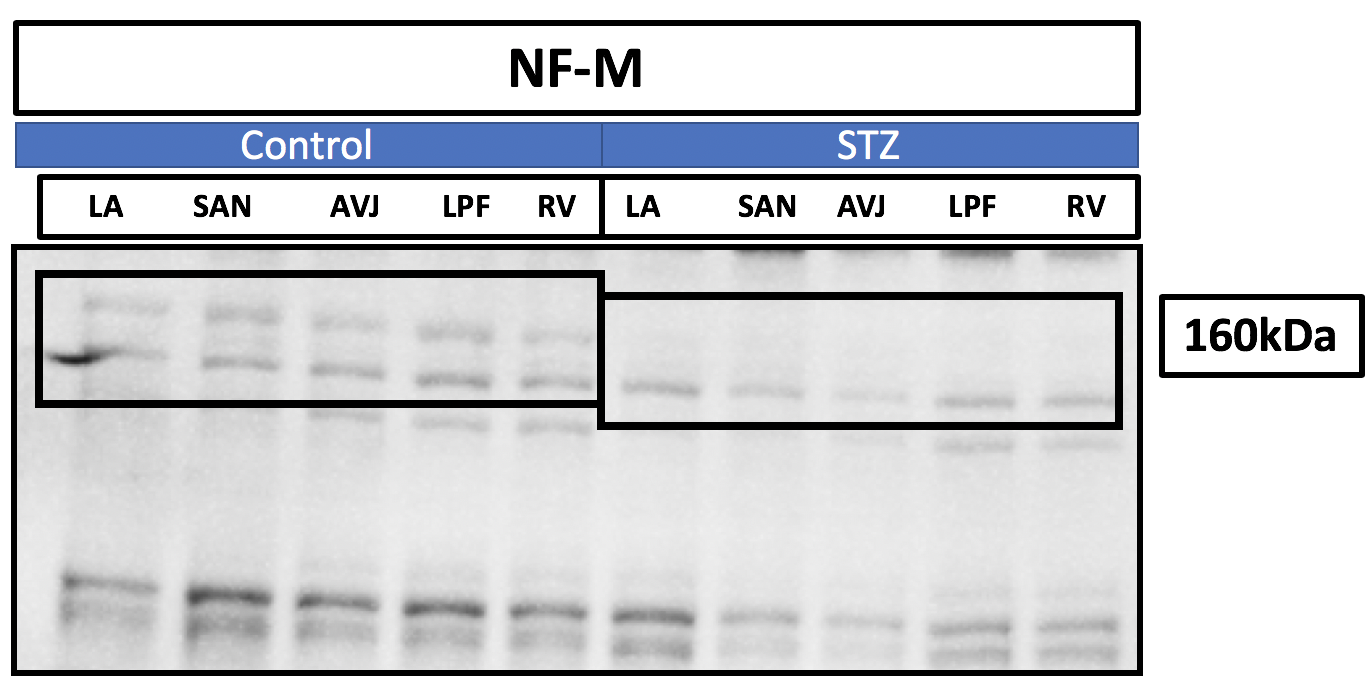


**Figure S17**: **Western blot membrane images of NF-M in LA, SAN, AVJ, LPF and LV for control and STZ induced diabetic rats**. Black borders around the images represent regions illustrated in Figure 6E for NF-M where specific bands for this protein were observed at ~160kDa.


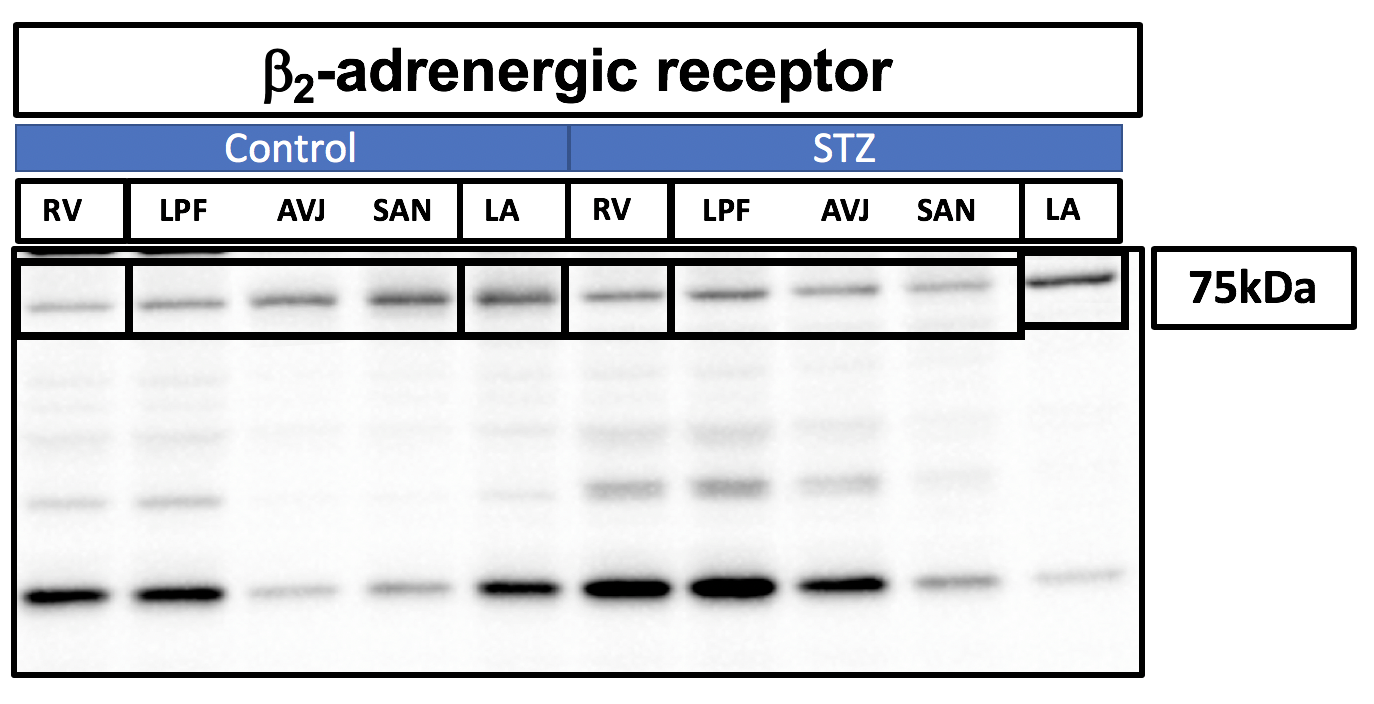


**Figure S18**: **Western blot membrane images of β_2_-adrenergic receptor in LA, SAN, AVJ, LPF and LV for control and STZ induced diabetic rats**. Black borders around the images represent regions illustrated in Figure 6E for β_2_-adrenergic receptor where specific bands for this protein were observed at ~75kDa.


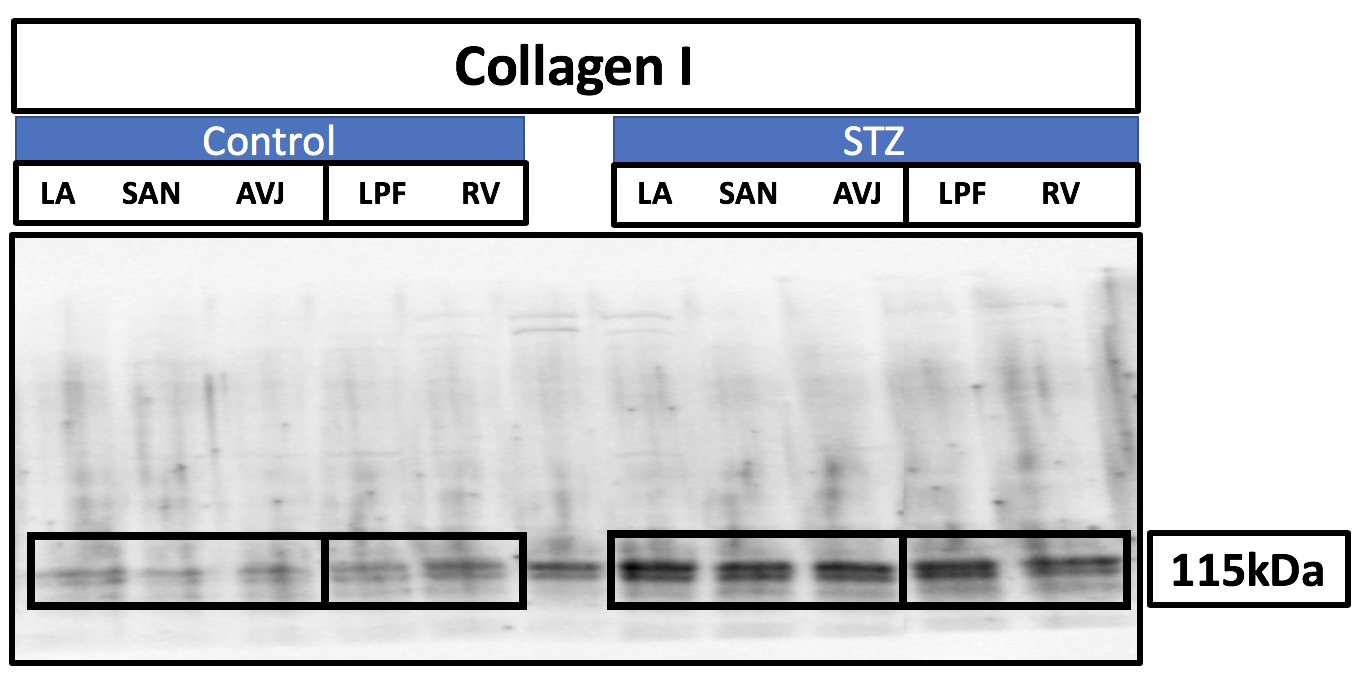


**Figure S19**: **Western blot membrane images of collagen in LA, SAN, AVJ, LPF and LV for control and STZ induced diabetic rats**. Black borders around the images represent regions illustrated in Figure 6E for collagen where specific bands for this protein were observed at ~115kDa.


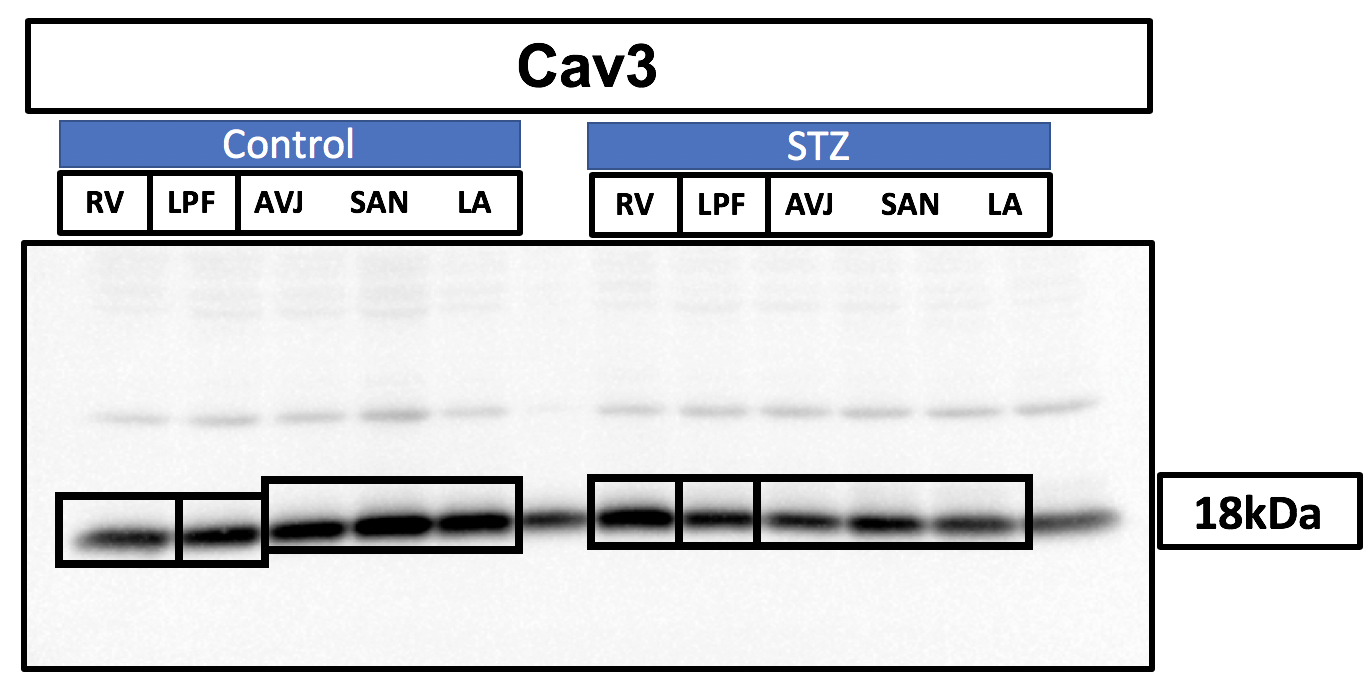


**Figure S20**: **Western blot membrane images of Cav3 in LA, SAN, AVJ, LPF and LV for control and STZ induced diabetic rats**. Black borders around the images represent regions illustrated in Figure 6E for Cav3 where specific bands for this protein were observed at ~18kDa.


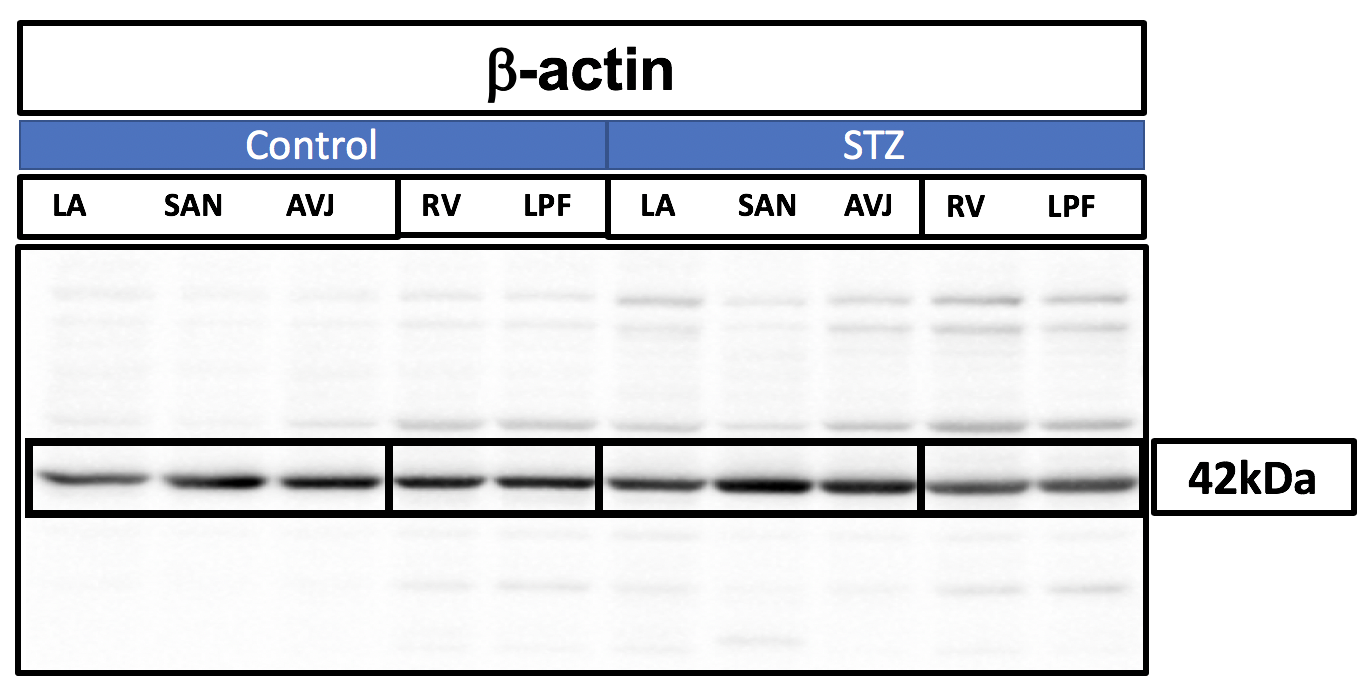


**Figure S21**: **Western blot membrane images of β-actin in LA, SAN, AVJ, LPF and LV for control and STZ induced diabetic rats**. Black borders around the images represent regions illustrated in Figure 6E for β-actin where specific bands for this protein were observed at ~42kDa.
